# Detection and pharmacokinetics of licochalcone A in brains of neuroinflammatory mouse model

**DOI:** 10.1007/s00210-025-04579-w

**Published:** 2025-09-08

**Authors:** Dalia Nour, Azza El-Azzhary, Rasha Hanafi, Reham M. Abdelkader

**Affiliations:** 1 Pharmacology and Toxicology Department, Faculty of Pharmacy and Biotechnology, German University in Cairo, Gamal Abdel Nasser, 11835 New Cairo, Egypt; 2 Pharmaceutical Chemistry Department, Faculty of Pharmacy and Biotechnology, German University in Cairo, Gamal Abdel Nasser, New Cairo, 11835 Egypt

**Keywords:** Cognitive dysfunction, Licochalcone A, Gas chromatography-mass spectrometry, Blood–brain barrier, Neuroinflammatory diseases

## Abstract

**Abstract:**

Licochalcone A (LCA), a natural flavonoid with potent anti-inflammatory properties, has shown promise as a neuroprotective agent. However, its ability to cross the blood–brain barrier (BBB) and exert central effects remains underexplored. In this study, we demonstrate for the first time that LCA enhances cognitive function in a lipopolysaccharide (LPS)-induced neuroinflammatory mouse model and effectively penetrates the BBB. Following intraperitoneal administration of LCA (20 mg/kg), behavioral assessments via novel object recognition and Y-maze tests revealed significant improvements in non-spatial and spatial short-term memory. To investigate whether LCA is able to cross the BBB, we first developed and validated a sensitive GC–MS/MS method (LOD, 0.14 µg/mL; LOQ, 0.42 µg/mL) capable of quantifying LCA in brain tissue. The method revealed LCA brain concentrations peaking at 4 h (*T*_max_), with sustained levels above 15 µg/mg up to 8 h post-injection. Notably, LPS-pretreated animals exhibited higher BBB permeability to LCA, suggesting inflammation-enhanced CNS penetration. This is the first report to confirm LCA’s brain permeability and provide pharmacokinetics in brain tissue. Our findings not only validate the central neuroprotective potential of LCA but also establish a reliable analytical method for the detection of LCA.

**Graphical Abstract:**

Licochalcone A (LCA) enhanced the cognition in the LPS-neuroinflammatory mouse model. To further understand its neuroprotective effect, a rapid sensitive GC–MS/MS bioanalytical method has been established to confirm LCA’s passage through the blood–brain barrier and perform a pharmacokinetics profiling in mice’s brain samples.
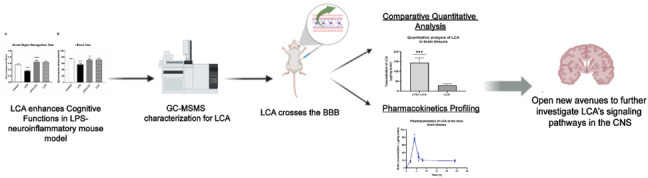

**Supplementary Information:**

The online version contains supplementary material available at 10.1007/s00210-025-04579-w.

## Introduction 

Cognitive decline is a cardinal feature of a variety of neurodegenerative diseases including Alzheimer’s disease (AD), Parkinson’s disease (PD), and Huntington’s disease (Panegyres 2004). Although aging is considered the primary risk factor for memory impairment and the onset of these neurodegenerative disorders, neuroinflammation is increasingly being identified as a potential mediator of these cognitive disorders (Franceschi et al. 2007; Kumar 2018; Mukhara et al. 2020). While inflammatory response initially plays a beneficial role in allowing tissue reconstruction in the presence of neuronal damage (Donnelly and Popovich 2008), when the neuronal insult persists, chronic inflammation develops gradually, taking its toll on our brains (Glass et al. 2010).

There has been a growing interest in the neuroprotective effects of flavonoids and natural products and in investigating their effectiveness in enhancing cerebral functions (Commenges et al. 2000; Letenneur et al. 2007; Müller et al. 2009). Licochalcone A (LCA), known chemically as (2E)−3-[4-Hydroxy-2-methoxy-5-(2-methylbut-3-en-2-yl)phenyl]−1-(4-hydroxyphenyl)prop-2-en-1-one, is a natural compound derived primarily from the roots of *Glycyrrhiza inflate* (Simmler et al. 2017). LCA belongs to the flavonoids class, a derivative of the chalcone skeleton, which is based on the [(E)−1,3-diphenyl-2-propene-1-one] structure. This chemical scaffold of chalcones consists of two aryl rings (A and B) connected by an α,β-unsaturated carbonyl system and is of medicinal chemistry importance (Thapa et al. 2021). It exhibits a wide range of pharmacological properties including antibacterial, antioxidant, bone protective, anticancer, blood glucose, and lipid level regulation (Quan et al. 2012; Ming et al. 2014; Shang et al. 2014; Cantelli et al. 2017; Li et al. 2022). The wide spectrum of LCA’s activity is believed to be associated with the α, β unsaturated carbonyl functional group in the chalcone structures, which serves as a potential Michael acceptor interacting with thiol groups like those in the cysteine residues (Dinkova-Kostova et al. 2001). LCA is recognized as a potent anti-inflammatory agent acting mainly through nuclear factor κB (NF-κB) and NRF2 signaling pathways in acute as well as chronic inflammatory conditions (Li et al. 2022). This has led to a growing interest in studying LCA’s neuroprotective effects. LCA has been shown to reduce the pro-inflammatory cytokines such as TNF-α and IL-1β (Chu et al. 2012a; Liu et al. 2018b) and inhibit microglia-mediated neuro-inflammation, specifically in lipopolysaccharide (LPS)-induced neurotoxicity (Huang et al. 2017a; Chiu et al. 2018). Furthermore, LCA has demonstrated its ability to prevent hippocampal neuronal degeneration, promote anti-apoptotic mechanisms, and alleviate cellular stress and neuro-inflammation in a kainic acid epilepsy animal model (Busquets et al. 2018). In this framework, LCA emerges as a promising candidate for investigation in neurodegenerative diseases, where neuroinflammation is a key factor, and its potential impact on cognitive functions.

Despite LCA’s significant potential in treating and preventing various clinical diseases, little research has been conducted regarding its analysis in biological tissues (Nadelmann et al. 1997a, b; Weng et al. 2019). LCA belonging to the oxygenated chalcones with two hydroxyl functional groups (–OH) in its structure is hardly volatile and would typically be considered a liquid chromatography (LC) application. While gas chromatography (GC) is limited to volatile compounds that possess thermal stability at the operating temperature, derivatization reactions can be employed to make non-volatile compounds like LCA amenable to GC analysis. Moreover, given GC’s strong performance in terms of reproducibility and resolution (Nolvachai and Marriott 2013), GC–tandem mass spectrometry (GC–MS/MS) emerges as a suitable technique for separation and detection, particularly when aiming for low detection limits in highly complex matrices.

Therefore, this study aimed to investigate the ability of LCA to enhance cognitive functions in an LPS-neuroinflammatory mouse model and, furthermore, to assess if the neuroprotective effects of LCA are due to its ability to pass the blood–brain barrier (BBB) and directly affect the central nervous system (CNS), or if it could be attributed to a peripheral effect. Accordingly, our second aim was to develop an analytical method for detecting and assessing the pharmacokinetics of LCA in mice brains. To that end, a sensitive and rapid bioanalytical method using GC–MS/MS was developed and optimized to assess the ability of LCA to pass the BBB in the brains of the in vivo model.

## Materials and methods

### Materials

Licochalcone A standard, (2E)−3-[4-hydroxy-2-methoxy-5-(2-methylbut-3-en-2-yl)phenyl]−1-(4-hydroxyphenyl)prop-2-en-1-one, was purchased from Cayman, USA. Icaritin standard ≥ 98% (HPLC grade), N, O-bis(trimethylsilyl) trifluoroacetamide (BSTFA), alkane standard mixture analytical standard (C10–C40 all even), lipopolysaccharide (LPS) (*Escherichia coli*, 055:B5), ethanol (LC–MS grade), and methanol (LC–MS grade) were provided from Sigma-Aldrich, Germany. Anhydrous pyridine, tert-butyl hydroquinone (TBHQ) 98% extra pure, and Tris buffer (AR grade) were supplied from Alpha Chemika, India.

### Animals

All studies were performed using adult male Swiss albino mice (6–8 weeks old), weighing 20–30 g. Mice were purchased from the Egyptian National Research Centre and transported under standard conditions. Upon arrival at the institutional animal facility, the animals were acclimatized for 7 days before any experimental procedures. All animal procedures were approved by the Ethics Committee of the German University in Cairo (PTX-2023–06-RA) and in accordance with the Care and Use of Laboratory Animals (8th edition) and the ARRIVE (Animal Research: Reporting of In Vivo Experiments) guidelines. Mice were group-housed (8/cage) in frequently cleaned and disinfected cages and were provided with water and dry food pellets ad libitum. The animal facility was subjected to 12 h of light/dark cycles with maintained constant temperature and humidity. The mice were allowed to acclimatize to the laboratory environment and facilities for at least 3 days before the commencement of the studies, to reduce stressors. Anesthetic exposure and heart perfusion prior to brain collection were avoided to minimize potential interference of anesthetics with bioanalysis (Slupe and Kirsch 2018; Spieth et al. 2021). Before conducting each experiment, the required sample size (*n*) was calculated to minimize animal suffering. For the scope of the study, 40 mice were used to conduct behavioral tests and bioanalysis, while another group of 30 mice was specifically utilized for evaluating pharmacokinetic parameters.

### LPS-neuroinflammatory mouse model

A neuroinflammatory mouse model induced by LPS was developed as described before by Lee et al. (2008a). This neuroinflammatory model was well-characterized previously by our research group, showing memory impairment, neuroinflammation, and neurodegenerative changes in the cortex and hippocampus (Zakaria et al. 2016; Abdel-Kader et al. 2021; Samir et al. 2023; Wagdy et al. 2023). For treatment, initial stock solutions of LCA (200 mg/mL) in DMSO and LPS in saline (1 mg/mL) were prepared. On each injection day, fresh injection solutions of LPS (25 µg/mL) by dilution with saline and LCA (2 mg/mL) by dilution with corn oil (DMSO: corn oil, 1:99) were prepared. Mice received LPS at a dose of (250 µg/kg) to induce neuroinflammation, a regimen based on previous studies demonstrating its effectiveness in eliciting neuroinflammatory markers and behavioral changes with repeated administration (Lee et al. 2008b; Katafuchi et al. 2012; Zhao et al. 2019). LCA was administered at 20 mg/kg, a dose supported by prior studies showing significant anti-inflammatory and neuroprotective effects without observable toxicity (Chu et al. 2012b; Hu and Liu 2016; Liu et al. 2018a; Shu et al. 2022).

Mice were divided randomly into four groups as follows: (i) control group, mice received vehicle injections (saline followed by DMSO/corn-oil vehicle); (ii) LPS onlygroup, LPS followed by the DMSO/corn-oil vehicle; (iii) treatment group, LPS followed by LCA; and (iv) drug control group, saline followed by LCA. For seven consecutive days (1–7 days), all animals received a first intraperitoneal (ip) injection of either LPS or saline; on days 4–7, a second injection (DMSO/corn-oil vehicle or LCA) was administered 2 h later. A 2-h interval was used between LPS injection and LCA treatment to allow for LPS-induced neuroinflammatory activation before therapeutic intervention. This timing also avoids potential direct pharmacological interaction and is consistent with previous post-LPS treatment protocols (Zakaria et al. 2016; Badshah et al. 2016).

**Fig. 1:**
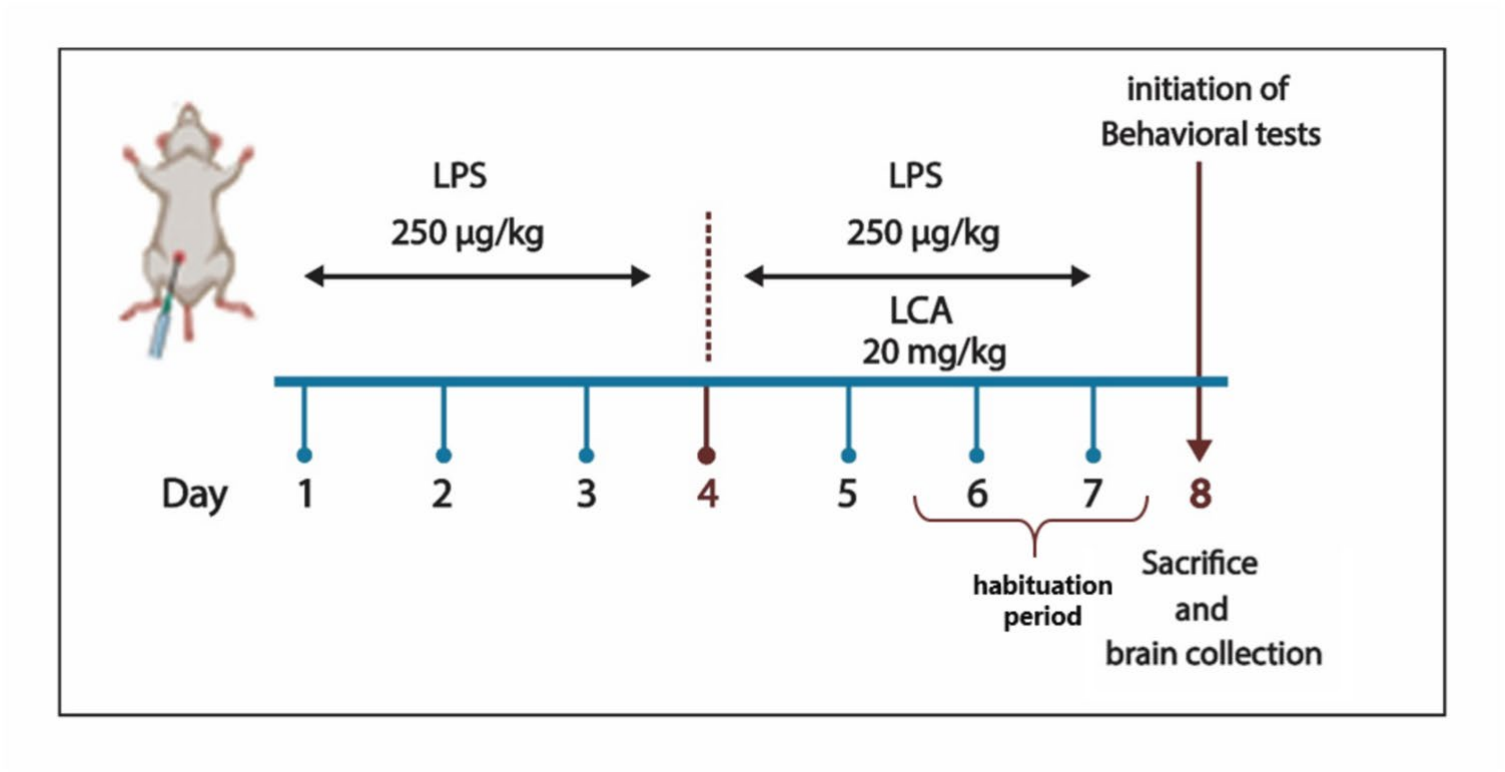
The treatment plan illustrated the establishment of the LPS neuro-inflammatory model and LCA treatment

The behavioral assessment was performed 24 h after the final dose (day 8), after which all mice were sacrificed, and their brains were collected for subsequent bioanalysis (Fig. 1).

### Behavioral tests

A total number of 40 mice were randomly assigned into four groups (*n* = 10 per group) before performing the behavioral experiments and kept blinded throughout the study until all data had been collected. The experimental unit in these assays was a single animal, and the primary outcomes were memory and cognitive functions. Mice underwent a 2-day habituation period spanning the last 2 days of the treatment regimen to avoid the potential influence of stressors on the animal’s behavior during the test. On days 6 and 7, mice were transported from their housing room to the behavioral testing room using the same route and procedures as on the actual test day and were allowed to acclimate in the testing room for several hours before being returned to their home cages.

All the behavioral tests were commenced in a quiet and dimly lit experimental room, distinct from the housing room. The order of mice being tested was randomized by the experimental group to avoid endocrinological stress responses between the within-cage mice (Lyte et al. 2005; Arndt et al. 2009). Mice failing to show exploratory behavior in the training phase were excluded from the study. The number of excluded animals did not significantly differ between groups, indicating comparable baseline exploratory activity.

### Novel object recognition (NOR) test

To assess non-spatial cognitive performance, the NOR test was conducted after completing the treatment regimen. Mice were habituated to handling for 2 successive days before the beginning of the test. NOR test was performed in a white wooden box with dimensions 40 cm × 40 cm × 60 cm (length × width × height) (German University in Cairo, Egypt) with discriminated objects and a video camera mounted on a tripod above it, in a way to provide full scope of the experimental arena, with a clear visualization of the object area, to record the movements of the mice. The objects used in the NOR test were wooden pyramids painted yellow, therefore brighter, and wooden cylinders painted dark blue, therefore darker. They were matched for texture and weight and used interchangeably as familiar or novel objects to prevent object-specific bias. A preliminary acclimatization period of 10 min was conducted to mitigate the influence of anxiety and stress on the study outcomes. During this habituation phase, mice freely explored the empty NOR arena for 10 min to acclimate to the environment and reduce novelty-related anxiety. The NOR test consists of two sessions, a training and test session, with an inter-trial interval of 2 h. The training session was during which each mouse was introduced to the experimental area and allowed to explore two identical sample objects for 10 min. The test session is where one of the sample objects was replaced by a novel one and each mouse was left to explore them for 5 min. The sample and novel objects, as well as the novel object place (i.e., right or left), were randomized between mice, to avoid any bias towards a specific object or a specific direction. The chamber and objects were cleaned with 70% ethanol to avoid olfactory stimuli from previous animals after each training session. Object interaction was defined as an event where a mouse’s head was within 2 cm of the object and directed towards the object. Sitting or leaning on the object was not considered object exploration (Ennaceur and Delacour 1988; Clark and Martin 2005). In the NOR test, mice with less than 7 s of total object exploration during either the training or test session were excluded from the analysis. The video recordings were analyzed blindly and used to calculate the discrimination ratio as follows:

$$\text{Discrimination ratio}=\frac{\text{Novel object interactions }(\mathrm{sec})}{\text{Total object interactions }(\mathrm{sec})}$$

### Y-maze spontaneous alternation test

The short-term spatial working memory and exploratory activity of the mice were assessed using the Y-maze spontaneous alteration test, according to the working protocol of Arai et al. (2001). The Y-maze apparatus consisted of three identical arms (arm length, 32 cm; height of wall, 10 cm, and 5 cm wide, extending from a central platform at 120°, German University in Cairo, Egypt) with a video camera mounted on a tripod above the Y-maze, to automatically record the movement and behavior of mice during the test. Each mouse was introduced to the test apparatus and allowed to freely explore the three arms for 8 min. The place of each arm was randomized between mice, but kept constant for the same mouse, to reduce any sort of bias related to the location of the arms. The sequences of animal’s entries in the arms were recorded. For an entry to be counted, the four paws and the tail of the animal have to be inside the arm. Mice with fewer than eight arm entries during the 5-min trial were excluded from analysis. Working memory was calculated based on the number of spontaneous alternations, which is defined as successive entries into three distinct arms consecutively without any repeated entry to any of the arms. The results were expressed as “spontaneous alternation percentage” using the equation: % spontaneous alternation = [Spontaneous alternations/(Total number of entries − 2)] × 100.

### Instrumentation

All Analytical procedures were performed using a GC–MS/MS on an Agilent 8890 A GC coupled to a 7010B triple quadrupole mass spectrometry (MS) (Agilent, CA, USA). GC–MS/MS is equipped with a split/splitless injection port and a 7693 A autosampler. The analytes were separated on a DP-5MS capillary column (30 m length, 0.25 mm inner diameter, 0.25 µm film thickness). Helium gas (grade 6.0) was used as the carrier gas at a constant flow rate of 1.6 mL/min through the column. Autosampler 7693 was used for injection and introducing samples into the column through a 10-µL syringe, injecting a volume of 1 µL. The mode of injection used was pulsed splitless with a pulse pressure of 60 psi and a purge time delay of 2 min. The oven temperature gradient program was as follows: hold an initial temperature of 200 °C for 1 min, ramp to 280 °C at 30 °C/min, then ramp to 300 °C at 2 °C/min, and finally hold for 2 min. All samples were injected in anhydrous pyridine solvent with a solvent delay time of 4 min. The column was reconditioned at 300 °C for 2 min to eliminate all impurities co-extracted from the matrix and minimize carry-over effects. The temperatures of the injection port and MS transfer line were 280 and 300 °C, respectively. The MS analyses were conducted in electron impact (EI) mode with a dual filament. An ionization energy at 70 eV was applied. The ion source temperature and MS quadrupole were set at 230 °C and 150 °C, respectively. For ion fragmentation, nitrogen (with helium quench gas) was used as the collision-induced dissociation (CID) gas. The mass spectrometer detector (MSD) was operated in full scan mode in a mass range of *m/z* 50–900 to identify the mass spectrum and retention times of the analyte and the internal standard (IS).

### Bioanalytical method development

#### Method optimization

The development of a proper gradient oven temperature program is a key factor in achieving good separation of the analyte peaks. The gradient oven temperature program and the run time were optimized using a mixture of derivatized reference standards for LCA and icaritin (IC), each at a concentration of 5 µg/mL, and injected into the GC–MS/MS system utilizing standard instrument settings. During this study, temperature ramps were tested, and the results in terms of short analysis time and good peak separation for the analyte and IS were analyzed.

#### GC–MS/MS characterization of the analyte

An identification approach according to the Food and Drug Administration (FDA) (Food and Drug Administration (FDA) and Center for Veterinary Medicine 2003; Rivier 2003) has been followed to confirm the identity of LCA in the biological samples, which in this study are the mice brain tissues.

- Sample screening

First, screening for LCA (analyte) and IC (IS) was performed by operating the MSD in a full scan mode in a mass range of *m/z* 50–900. For this purpose, reference standards were derivatized and analyzed at a concentration of 5 µg/mL to investigate the spectral data matching the fragmentation pattern and the nominal molecular weight for the derivatized form of the analyte and IS, then documenting their specific retention time.

- Multiple ion monitoring (MRM) transitions

Multiple reaction monitoring (MRM) transitions were developed and optimized for both LCA and IC derivatives. First, parent ions were determined using MSD in full scan mode, and the most unique and abundant ions were chosen for both the analyte and the IS. Second, product ions were determined by operating the MSD in product ion scan mode with variable CEs in the range of 3–42 V (3 V step), and then three characteristic product ions with high relative abundance were chosen. Third, the (GC–MS/MS Optimizer for GC-TQ) software was employed to optimize the collision energy (CE) and dwell time for each transition.

- Qualitative analysis and confirmation of identity

Identity confirmation was performed according to the criteria used for the first identification of the analytes per the FDA, including retention time (RT) and three MRM diagnostic transitions. The RT and the relative abundance of MRM diagnostic ions of the spiked analyte shall not vary within ± 1% and ± 20%, respectively, from that of the reference standard analyzed in the same batch (Food and Drug Administration (FDA) and Center for Veterinary Medicine 2003; Rivier 2003).

The confirmation process approach was as follows, using the MSD in MRM mode:

1. Blank control samples, including pyridine solvent and blank brain samples (pooled brain homogenates collected from untreated mice), were analyzed and confirmed to be true negatives.
2. Spiked brain samples with both LCA and IC derivatives were analyzed and confirmed to meet the previously mentioned identification criteria.
3. System suitability was established by injecting sufficient replicates of the reference standards of both LCA and IC.
4. Blank brain samples were analyzed after a spiked sample to exclude a carry-over effect, resulting in a false positive result.

#### Kováts retention index

The Kováts retention index is a dimensionless measure indicative of the relative retention time of a compound in the chromatographic column, normalized to the retention time of a reference compound (Zenkevich 2020). To determine Kovats Index (I), n-alkane mixture C_10_–C_40_ was used as the reference compound to normalize IC’s and LCA’s retention times using the following formula:

$$I=100\times \left[n+\frac{{\mathrm{log}t}_{x}-{\mathrm{log}t}_{n}}{{\mathrm{log}t}_{n+1} - {\mathrm{log}t}_{n}}\right]$$

where *n* is the number of carbon atoms in the tailing n-alkane, *t*_*x*_ is the retention time of the analyte, and *t*_*n*_ and t_*n*+1_ are the retention times of the tailing and heading n-alkane, respectively.

### Preparation of stock standards and working solutions

Main stock solutions were prepared for LCA (500 µg/mL) in methanol and IC (100 µg/mL) in ethanol and stored at 4 °C until use. Working solutions were freshly prepared for LCA at concentrations of 50 µg/mL and 5 µg/mL and for IC at a concentration of 15 µg/mL from the main stock solutions by dilution with methanol and ethanol, respectively.

### Preparation of calibrators and quality control (QC) samples

In addition to a blank and a zero calibrator (blank + IS), six non-zero calibrators were generated covering a concentration range of 0.5, 0.75, 1.5, 5, 10, and 20 µg/mL. The calibration solutions were prepared in triplicate (*n* = 3) by spiking 300 µL of the brain homogenate with the appropriate volumes from the freshly prepared LCA working solutions to reach the desired concentrations. Quality control (QC) samples were prepared in a manner similar to that used to prepare the calibrators at three concentration levels within the linear range of the assay: low (LQC), 0.5 µg/mL; medium (MQC), 7.5 µg/mL; and high (HQC), 15 µg/mL. The concentration of the IS in both the calibrators and QC samples was maintained at 0.5 µg/mL. Both the calibrators and QC samples were then extracted and derivatized following the sample preparation protocol.

### Method validation

Method validation has been performed according to the US FDA Bioanalytical Method Validation Guideline and the International Conference on Harmonization ICH Guideline M10 on Bioanalytical Method Validation (Food and Drug Administration (FDA) et al. 2018; European Medicines Agency (EMA) 2022). Under the optimized conditions, several parameters relevant to a quantitative and a qualitative method, including selectivity and specificity, linearity, limit of detection (LOD) and quantification (LOQ), accuracy, precision, and recovery, were evaluated. Additionally, matrix effects were also assessed per the European Medicines Agency guidelines for validation (European Medicines Agency 2015). The brain matrix used for method validation consisted of pooled brain homogenates collected from ten untreated mice.

#### Selectivity and specificity

Selectivity of the method was demonstrated by analyzing blank samples of the intended biological matrix, in this study brain matrix, to detect any potential interfering substances with the analyte and internal standard. Retention times of the extracted blank brain samples and those of the spiked brain samples with the analyte and IS were compared. If any interferences were observed, the signal of their peak area should be less than 20% of the peak area of the lower limit of quantification (LLOQ) and less than 5% of the average peak area of IS in blank samples.

#### Linearity

Ensuring the linearity of the developed method is a prerequisite parameter for the validation process. The linearity was assessed over the concentration range of 0.5–20 µg/mL from nine independent assays over three consecutive days (three calibration runs per day). A six-point calibration curve was constructed by plotting the peak area ratio (LCA peak areas relative to the corresponding IC (IS) peak area) versus the analyte concentration. The least regression method and the squared correlation coefficient (*r*^2^) were used to estimate linearity.

The calibration curve should meet the acceptance criteria, with a minimum of 75% of the calibrators falling within ± 15% of their nominal concentrations and the LLOQ not deviating more than ± 20% from its nominal concentration.

#### Limits of detection (LOD) and quantification (LOQ)

The limit of detection and limit of quantification were defined as LOD = 3.3 *σ*/*S* and LOQ = 10 *σ*/*S*, where *σ* is the standard deviation of the response and *S* is the slope of the calibration curve. The background response and the *S* were measured as the standard deviation of the *y*-intercept and the average slope, respectively, from nine independent assays.

#### Accuracy and precision

Accuracy and precision were evaluated using replicates of QC samples at LQC, MQC, and HQC levels of LCA spiked on pooled brain homogenates. Precision was measured as the coefficient of variation (CV%) from three independent assays within one experimental day (intra-day) or independent assays conducted over three consecutive days (inter-day). Accuracy was represented by the mean recovery from three independent assays across three consecutive days. The acceptance criteria for precision and accuracy require values to be within ± 15% CV or from nominal concentrations, and the LLOQ not deviating more than ± 20% CV or from its nominal concentration, respectively.

#### Recovery

The recovery of LCA in brain tissue was evaluated at the three QC concentrations: LQC, MQC, and HQC (*n* = 3 for each concentration). Extraction recoveries were calculated by comparing the (analyte-to-IS) peak area ratio of the spiked and extracted blank brain samples to the corresponding (analyte-to-IS) peak area ratio of the post-extracted brains spiked with the standards.

#### Matrix effect

The matrix effect was studied to evaluate the suppression or the enhancement of the analyte or the internal standard signals caused by the influence of the sample matrix. For this purpose, three QC levels at LQC, MQC, and HQC were prepared in triplicate and spiked on the solvent (pyridine) as well as on post-extracted brain samples. The matrix factor (MF) was determined by comparing the mean peak area of the analyte-spiked solvent and the post-extracted brain matrix as follows:

$$\mathrm{MF}=\frac{\text{Mean peak area for the matrix based analyte}}{\text{Mean peak area for the solvent based analyte}}$$

The IS normalized MF was determined as a ratio of the matrix factor of the analyte and the IS as follows:

$$\text{IS normalized MF}= \frac{\text{MF of the analyte}}{\text{MF of the IS}}$$

For each matrix lot evaluated, the accuracy should be within ± 15% of the nominal concentration, and the precision (CV%) should not be greater than 15%. The IS normalized MF should fall within 0.8–1.2.

### Biological sample preparation for analysis

#### Sample collection

Mice were sacrificed by cervical dislocation, and their brains were collected and separated from the cerebellum. The collected brains were washed with ice-cold Tris buffer (5 mM, pH 7.4) and then homogenized with 1 mL of the Tris buffer/100 mg brain using the 5-mL Dounce homogenizer. The brain homogenates were stored at − 80 °C till analysis. As a limitation, our current sample collection and extraction protocol does not specifically separate brain parenchyma from the vascular system. The homogenization included the entire brain components, allowing for a comprehensive analysis of LCA in the samples.

#### Sample extraction

Brain homogenates were removed from the − 80 °C freezer and allowed to come to room temperature. Brain matrix extraction was performed as described before (Paulke et al. 2006) with slight modifications. Briefly, in tightly sealed polypropylene tubes, 300 µL brain homogenate was mixed with 300 µL methanol, 200 µL tert-butyl hydroquinone (TBHQ) solution (5 mg/mL), and 200 µL HCl (1 M). The reaction mixture was vortexed for 1 min and inserted into a heat block at 90 °C for 2 h. The reaction tubes were then centrifuged at 3700 × *g* for 10 min, and the supernatant was collected in micro-centrifuge tubes and transferred for derivatization.

#### Sample derivatization

**Fig. 2:**
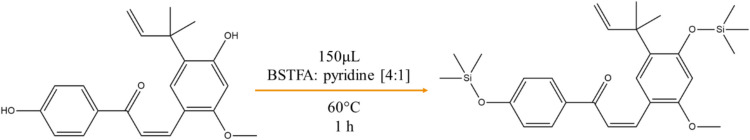
Derivatization reaction of LCA with N,O-bis(trimethylsilyl)trifluoroacetamide (BSTFA) in the presence of pyridine (4:1, v/v) to form the 2-TMS derivative. The reaction was performed at 60 °C for 1 hour to enhance volatility and thermal stability of the analyte for GC-MS/MS analysis

The chosen method for derivatization in this study involves silylation, utilizing the N, O-Bis(trimethylsilyl) trifluoroacetamide (BSTFA) as a silylating agent. The derivatization reaction is employed to produce silyl derivatives of the analyte, which are more volatile and more thermally stable. The reaction replaces active hydrogens with trimethylsilyl groups (TMS) through SN2 nucleophilic attack (Halket and Zaikin 2003). Extracted samples or reference standards were first placed in a vacuum concentrator for 2 h before performing the derivatization reaction to ensure complete dryness. Dried sample residues were derivatized by adding 150 µL of the mixture of BSTFA and pyridine in a (4:1) ratio into the samples’ tubes. The derivatization reaction was performed at 60 °C for 1 h (Fig. 2). The tubes were centrifuged at 21,460 × *g* for 1 min and transferred to glass vials suitable for GC–MS/MS analysis.

### Qualitative and quantitative analysis

Mice were sacrificed after the treatment regimen and behavioral testing for brain tissue collection to assess LCA’s permeability across the BBB and measure its brain levels (*n* = 6 per group, a total of four groups). For both qualitative and quantitative analysis on mice brain samples, the MSD was operated in time-segmented MRM mode for the selected transition ions.

### In silico ADME and BBB permeability predictions

In silico predictions of physicochemical properties and BBB permeability for licochalcone A (LCA) and its major metabolites were performed using two publicly available platforms: SwissADME (version accessed at http://www.swissadme.ch/) and admetSAR 1.0 (https://lmmd.ecust.edu.cn/admetsar1). The canonical SMILES structures of LCA and its metabolites were input into both platforms to calculate key ADME parameters, including cLogP**,** topological polar surface area (TPSA**)**, and predicted logBB values**.** BBB permeability probabilities were obtained using the BBB penetration models in admetSAR. Additionally, P-glycoprotein (P-gp) substrate predictions were recorded to assess the likelihood of efflux-mediated transport limitation. These computational predictions were used to support the interpretation of experimental data and to propose potential mechanisms of CNS exposure for LCA and its metabolic derivatives.

### Pharmacokinetics study

For pharmacokinetic analysis, 30 adult male Swiss albino mice were randomly divided into six groups (*n* = 5 for each group), according to sample collection times. Mice received a daily ip dose of LPS at 250 µg/kg for seven consecutive days and a single ip dose of LCA at 20 mg/kg at day 8. Mice were sacrificed, and brain tissues were collected after 0, 2, 4, 6, 8, and 24 h post-dosing and were then kept at − 80 °C before sample treatment. Collected brain samples were then extracted and derivatized as described previously and then further analyzed using the GC–MS/MS in MRM mode using the established MRM transitions. Pharmacokinetic parameters were determined by non-compartmental analysis using PK solutions software, version 2.0 (Summit Research Services).

### Statistical analysis

Data analysis and visualization were performed using GraphPad Prism (version 8.4.3, San Diego, CA, USA). For sample size (*n*) determination, a priori analysis was performed using the one-factor ANOVA test with a 0.05 significance threshold, power of 0.8, and effect size of 0.56 based on similar previous behavioral studies on the LPS-neuroinflammatory mouse model (Mostafa et al. 2018; Wagdy et al. 2019; Zhao et al. 2019; Abdel-Kader et al. 2021; Samir et al. 2023) and of 0.74 based on previous pharmacokinetics assays on the same mouse model (Santibáñez et al. 2022). All the measurements were taken from distinct samples. Residuals were analyzed for normal distribution using the Shapiro–Wilk and D’Agostino–Pearson omnibus normality test. The variance was assessed using the unpaired *t*-test and one-way ANOVA test, followed by the Sidak test. Data are given as arithmetic means ± SD. The level of significance was set at *p* < 0.05. *p*-values less than 0.05 were considered statistically significant (i.e., ∗∗∗*p* < 0.001, ∗∗*p* < 0.01, ∗*p* < 0.05), while ns = statistically not significant.

## Results

### Effect of LCA on memory in the LPS-neuroinflammatory mouse model

**Fig. 3:**
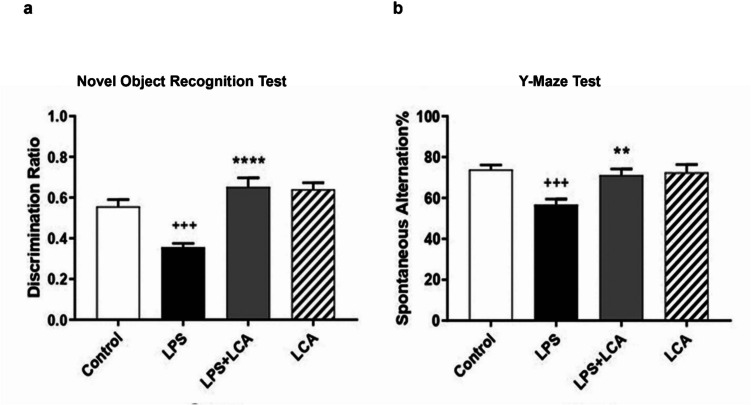
LCA improves memory and cognitive functions in the LPS-neuroinflammatory mouse model (**a**) LCA enhances the behavioral recognition memory of the LPS mouse model by increasing the discrimination ratio. Data are expressed as mean ±SEM, (*n *= 8-10 mice) (**b**) LCA improves the short-term working memory of the LPS-mouse model by increasing the spontaneous alternation percentage. Data are expressed as mean ±SEM, (*n* = 9-10 mice). ^+^*p *< 0.05, ^++^*p *< 0.01, ^+++^*p *< 0.001 relative to the control group and *p < 0.05, **p < 0.01, ***p < 0.001 relative to the LPS group

To investigate the impact of LCA on memory and cognitive functions in relation to neuroinflammatory disorders, behavioral tests were conducted on the LPS-neuroinflammatory mice model. Both the non-spatial form of recognition memory and the spatial short-term memory were profiled for the different treatment groups through NOR and Y-maze tests, respectively. The discrimination ratio in NOR testing reflected the mice’s ability to recognize a familiar object from a newly introduced novel object. In the Y-maze test, spontaneous alteration % served as a measure of exploratory behavior in the novel arm, indicative of intact memory. Notably, LPS reduced both the discrimination ratio and the spontaneous alteration % in mice when compared to control groups, suggesting damaged cortical and hippocampal functions, consistent with previous studies (Winters et al. 2004; Cohen et al. 2013). However, LCA treatment restored the explorative behavior (*p* < 0.01) and significantly enhanced the recognition memory (*p* < 0.001) of the familiar object from the new ones in treated mice groups (LPS + LCA) when compared to the LPS-treated groups. It is noteworthy that mice treated solely with LCA did not show alteration in non-spatial and spatial short-term memory compared to control groups (Fig. 3).

### Bioanalytical method optimization

Prior to any screening or identification analysis, the chromatographic parameters of the developed bioanalytical method were optimized, including the oven temperature gradient program, to obtain high sensitivity and good separation of the analytes within a short analysis timeframe. The initial attempt at optimizing the oven temperature gradient involved holding the initial oven temperature at 70 °C for 1 min, ramped to 300 °C at a rate of 5 °C/min, and then a final hold for 2 min at 300 °C. Although this temperature gradient program exhibited good separation and resolution power, it concurrently resulted in an extended total runtime of 49 min. After several trials, the temperature gradient was finally fine-tuned to the following specifications: a hold at an initial temperature of 200 °C for 1 min, ramp to 280 °C at 30 °C/min, then ramp to 300 °C at 2 °C/min, and finally hold for 2 min. This adjustment resulted in a significantly reduced total runtime of 15.6 min while maintaining optimal separation and resolution of analytes (Supplementary Figure S1).

### GC–MSMS characterization of derivatized LCA and icaritin

#### Screening reference standards in full scan mode

**Fig. 4:**
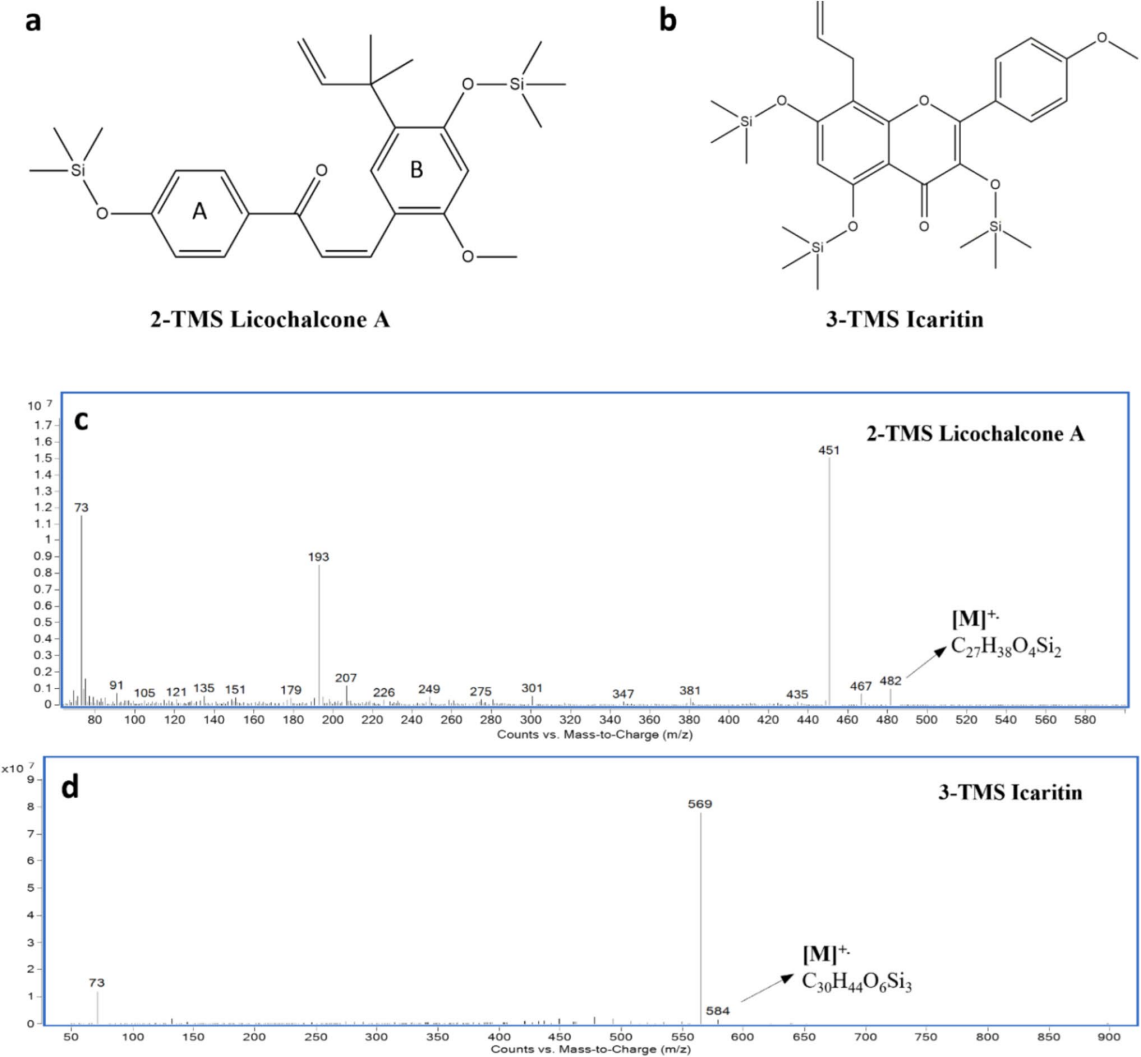
Chemical structures and +EI full scan mass spectra (range *m/z* 50-900) for the derivatized LCA and IC reference standards. (**a**) 2-trimethylsilyl Licochalcone A derivative (**b**) 3-trimethylsilyl Icaritin derivative (**c**) +EI mass spectrum for 2-TMS LCA at both 10.61 min and 11.22 min (d) +EI mass spectrum for 3-TMS IC at 10.04 min

GC–MS characterization for LCA has not been reported before in the literature; thus, preliminary screening for the target analyte is needed as a first step. This was achieved by screening reference standards for both compounds, operating the MSD in a full scan mode from range *m/z* 50–900. Two distinct eluting peaks denoted as *D*_1_ (10.6 min) and *D*_2_ (11.2 min) were identified as the 2-TMS LCA derivative (*Z*- and *E*-isomers, respectively), and eluting at 10.04 min was identified to be the 3-TMS IC derivative (Supplementary Figure S2). This identification was grounded on the presence of the parent ions corresponding to 2-TMS LCA at 482 *m/z* and 3-TMS IC at 584 *m/z,* detected in the EI mass spectrum for both derivatives (Fig. 4). Only the *D*_2_ peak for LCA was analyzed in our method because it represents the predominant, thermodynamically stable *E*-isomer and provides a consistent signal, while *D*_1_ appeared inconsistently and overlapped the internal standard’s retention window. All procedures were conducted under subdued light to minimize ex vivo isomerization. RTs, Kováts index, and characteristic ions for both the LCA and IC derivatives are summarized in Table 1.

**Table 1:** Molecular weight, RTs, Kováts Index, and characteristic diagnostic ion for the analyte and the IS derivatives

Compound	Molecular weight	Class	RT (min)	Kováts Index	Characteristic ions (m/z)	Absolute Abundance
*2-TMS* *LCA*	482	Analyte	11.22	2052.37	451 73 193	15016389 11509938 8530037
*3-TMS* *Icaritin*	584	Internal standard	10.04	2005.54	569 73	77863488 11871075

#### MRM transitions’ optimization

The MS/MS parameters were optimized to ensure the selectivity of the developed method. MRM transitions were established for both 2-TMS LCA and 3-TMS IC derivatives along with optimized CE and dwell time. The most unique and abundant ions, 451 and 569 *m*/*z*, were chosen as precursor ions for LCA and IC derivatives, respectively. After the product ion scan, the most abundant product ion was chosen as the quantifier ion, and the other two most intense were carefully chosen as the qualifier ion (Table 2).

**Table 2:** Quantifier and qualifier MRM transitions and the relative abundance ratio of quantifier to qualifier (RA)

Compound	Quantifier		Qualifier		RA
	Transition (*m/z*)	CE (V)	Transition (*m*/*z*)	CE (V)	
**2-TMS LCA**	451→347	33	451→435 451→421	33 43	100/73 100/44
**3-TMS icaritin**	569→554	33	569→437 569→343	43 42	100/89 100/53

A time-segmented MRM scan mode was established based on the specific time windows where the LCA and IC derivatives appear and on the previously established MRM transitions (Supplementary Figure S3). The MSD was operated to detect the three diagnostic MRM transitions for 2-TMS LCA derivative at two time segments, from 4 to 9.5 min and then from 11 to 15.6 min. Then, at a time segment from 9.5 to 11 min, the MSD was operated to detect the three MRM transitions specific for the 3-TMS IC derivative.

#### Identity confirmation in spiked brain samples

To confirm the identity of LCA and IC derivatives on spiked brain samples, the MSD was operated in the previously established time-segmented MRM mode, monitoring the three diagnostic MRM transitions for each analyte. Blank brain samples were first injected and confirmed to be true negatives of both analytes. Afterward, spiked brain samples with LCA and IC were injected into the GC and were confirmed to meet the confirmation criteria, RT, and MRM transitions’ intensities within ± 1% and ± 20%, respectively, compared to the reference standards (Supplementary Figure S4).

### Method validation

The developed bioanalytical method has been validated for licochalcone A, the analyte of interest. The validated method was able to selectively detect LCA in spiked and extracted brain samples, using 300 µL of brain homogenate and within a short analysis time of 15.6 min.

#### Selectivity and specificity

Blank brain samples showed no interference at the retention times of the analyte (licochalcone A) and the IS (icaritin). Additionally, the blank brain matrix showed no potential interfering substances (endogenous matrix components or metabolites) with the analyte and IS (Supplementary Figure S5). The consistent peak shape and retention time of LCA between the extracted and post-extracted brain matrices demonstrate the method’s reliability in terms of selectivity and specificity.

#### Linearity

A six-point calibration curve was constructed based on data from nine independent assays over three consecutive days. Good linearity was obtained for licochalcone A over the range of 0.5–20 µg/mL in the pooled control brain matrix. The calibration equation was *y* = 8.5133*x* + 1.7962 with *R*^2^ > 0.995. The calibration curves were continuous and reproducible with the coefficients of variation (CV%) ranging between 0.51 and 6.39% and the percent accuracies ranging between 89.54 and 111.83%. (Supplementary TableS1).

#### Limits of detection (LOD) and quantification (LOQ)

The limit of detection (LOD) for LCA in spiked brain matrix was estimated to be 0.14 µg/mL corresponding to the signal-to-noise ratio 3:1. The limit of quantification (LOQ) was determined to be 0.42 µg/mL corresponding to the signal-to-noise ratio 10:1. LOD and LOQ values were calculated based on the standard deviation of the response and the slope of the calibration curve in accordance with ICH Guideline M10 on Bioanalytical Method Validation (Food and Drug Administration (FDA) et al. 2018; European Medicines Agency (EMA) 2022). The method demonstrated high sensitivity, with a signal-to-noise ratio (4.2) at the lowest tested concentration.

#### Accuracy and precision

The results showed that the developed method exhibits satisfactory accuracy and precision. The accuracy and precision values for both the intra- and inter-day assays fell within the acceptable range of ± 15% for MQC and HQC and ± 20% for LQC. The coefficients of variation (CV%) for the intra- and inter-day precision ranged between 3.05 and 6.17% and 2.69 and 11.15%, respectively. Percent accuracies were within 92.76–112.83%, except for LQC was within 89.56–119.36% for intra-day assays. While inter-day percent accuracies ranged between 92.76 and 114.78%, except for LQC ranged between 89.56 and 105.73%. The mean values for inter- and intra-day accuracy and precision are summarized in Table 3.

**Table 3:** The intra- and inter-day measured concentrations, precision, and accuracy values for quantification of LCA at 3 QC level concentrations. Data expressed as mean ± SD, *n* = 5

Spiked concentration (µg/mL)	Measured concentration (µg/mL)	Precision (CV%)	Accuracy (%)
Intra-day			
0.5	0.51 ± 0.06	6.17	101.33
7.5	7.15 ± 0.19	3.38	95.34
15	16.12 ± 0.79	3.05	107.48
Inter-day			
0.5	0.49 ± 0.03	11.15	97.21
7.5	7.22 ± 0.24	2.69	96.29
15	16.59 ± 0.51	4.89	110.61

#### Recovery

The extraction recovery was assessed for licochalcone A from the brain matrix. The extraction recovery values fell within the range of 63.28–72.04% and were observed to be reproducible with CV% values ranging between 0.85 and 4.78%. Mean recovery values were summarized in Table 4.

**Table 4:** Mean recovery and matrix effect for LCA in the brain matrix. Data expressed as mean ± SD, *n* = 3

Spiked concentration (µg/mL)	Recovery		Matrix effect		
	%Recovery	CV%	Matrix factor	IS normalized matrix factor	CV%
0.5	67.80 ± 2.01	2.97	0.44 ± 3.82	0.91 ± 0.05	5.41
7.5	63.78 ± 0.54	0.85	1.82 ± 2.51	0.87 ± 0.07	8.41
15	68.48 ± 3.27	4.78	1.69 ± 3.48	0.86 ± 0.03	3.49

#### Matrix effect

Since the intended matrix for analysis is a complex biological matrix (brain tissues), the matrix effect was studied to verify the suppression or enhancement of the signals. The IS normalized MF for LCA ranged between 0.81 and 0.97 in the brain matrix, and the CV% values were within 3.49–13.59%. The results obtained showed that the matrix effect is within the permissible range with slight signal suppression. Values less than 1 indicate suppression, while values greater than 1 indicate enhancement of the detector signal. Mean values for the matrix factor and the IS normalized matrix factor are summarized in Table 4.

### Application of the analytical method

#### Permeability of LCA across BBB

**Fig. 5:**
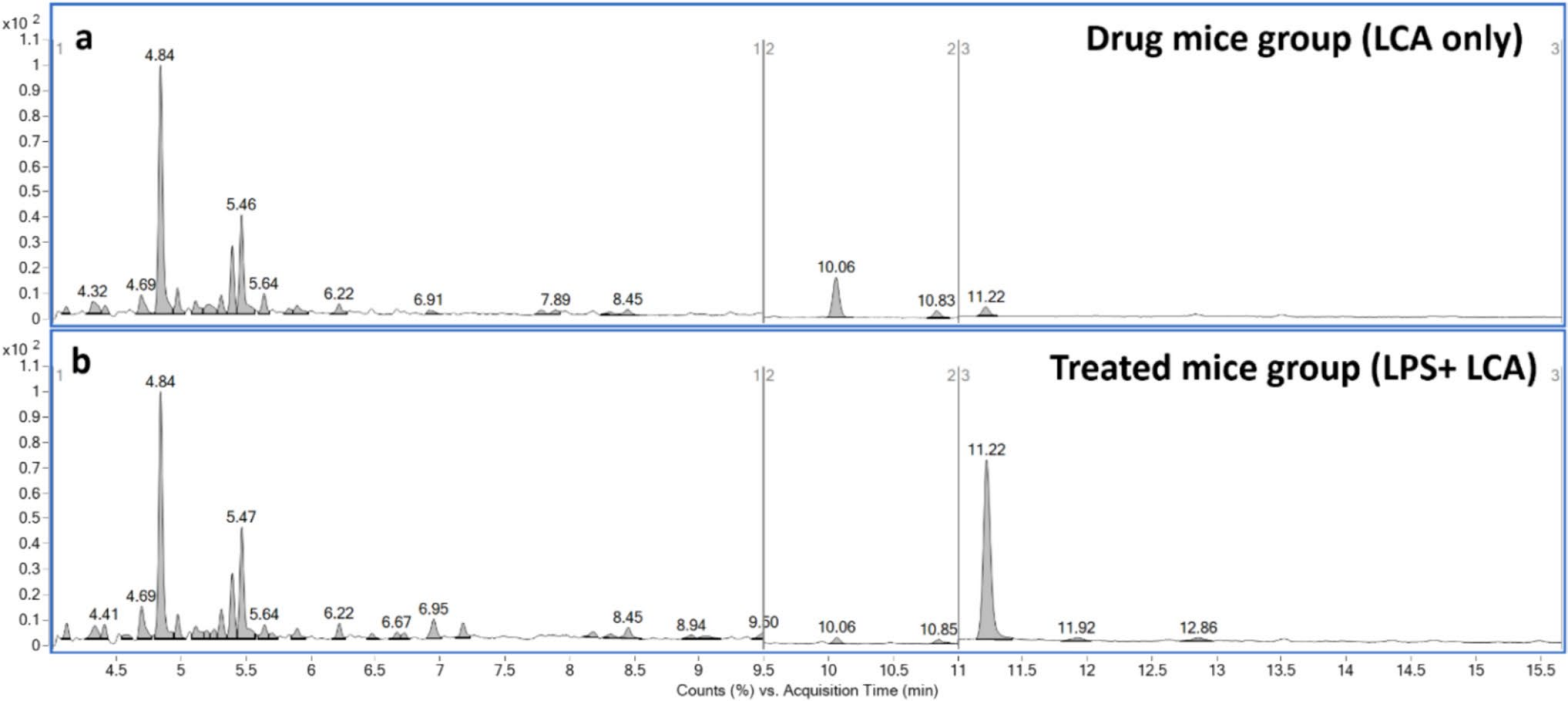
LCA crossed the BBB in mice brains of the LPS-neuroinflammatory mouse model. (a) +EI-MRM chromatogram for drug control mice group (b) +EI-MRM chromatogram treated mice group (LPS+ LCA). 2-TMS LCA derivative peak appeared at 11.22 min in both chromatograms

After the validation process, the utility of the developed GC–MS/MS bioanalytical method was demonstrated by assessing the LCA’s ability to cross the BBB in the context of neuroinflammatory disorders. After the treatment regimen, mice brain samples were collected, subjected to sample extraction and derivatization, and then transferred into GC vials for analysis. First, mice brains from control mice groups (received vehicle only) and LPS groups (received LPS + drug’s vehicle) were analyzed and confirmed to be true negatives of the analyte (Supplementary FigureS6). Subsequently, brains from the treated mice group (received both LPS and LCA) and drug control groups (received LCA’s vehicle + LCA) were analyzed, confirming the presence of LCA peak at its specific RT of 11.22 min, proving its capability to pass the BBB (Fig. 5).

**Fig. 6:**
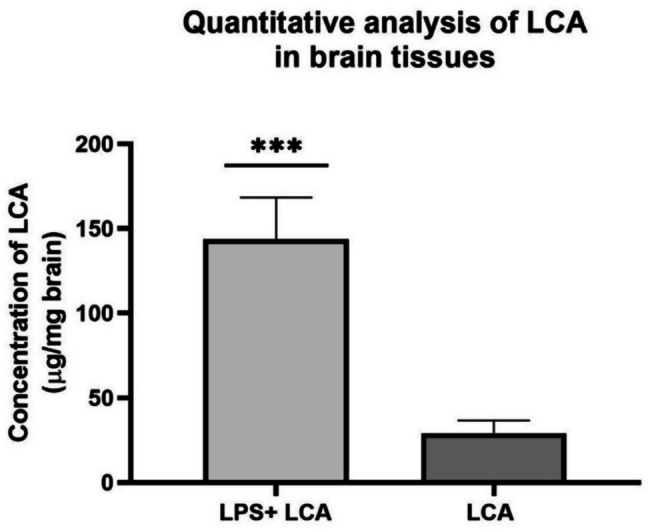
Quantitative analysis for LCA in brain tissue of the LPS neuro-inflammatory mice model. Treated mice groups (LCA+ LCA) showed significantly higher concentrations (143.68 ±24.82 µg/mg brain tissue, *n *= 6 brain samples from 6 mice) vs Drug control mice groups (LCA only) (29.07 ±7.58 µg/mg brain tissue, *n *= 6 brain samples from 6 mice) (*p* < 0.0001). Data represent means ± SD, ***p < 0.001, **p < 0.01, *p < 0.05

Notably, a greater LCA permeation through the CNS was observed in treated mice groups (LPS + LCA) than in drug control mice groups. This observation signifies the impact of LPS in impairing BBB function and augmenting its permeability. This difference in permeability observed was further verified through quantitative analysis comparing the levels of LCA within the brain tissues conducted between the treated and drug control mice groups in the LPS neuro-inflammatory mouse model (Fig. 6). The average concentration of LCA in the treated mice groups (LPS + LCA) was determined as 143.68 ± 24.82 µg/mg brain tissue (*n* = 6), in contrast to LCA concentration in the drug control mice groups, which measured 29.07 ± 7.58 µg/mg brain tissue (*n* = 6) (*p* < 0.0001).

#### Pharmacokinetic study

**Fig. 7:**
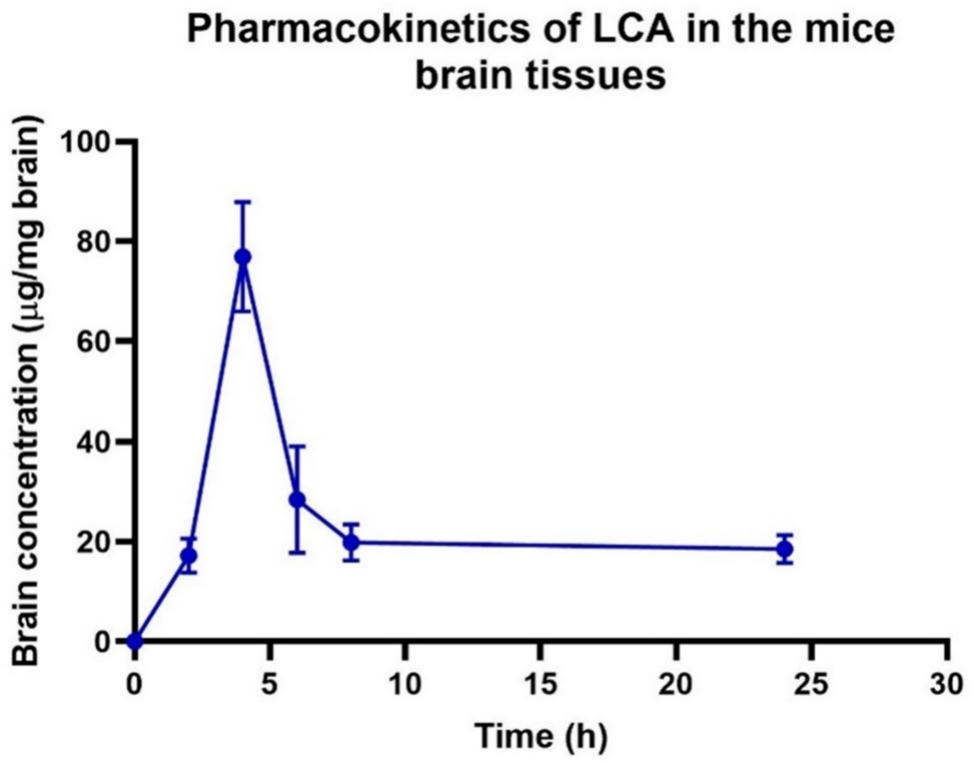
Brain concentration-time curve of the pharmacokinetic study for LCA in mice brain tissues after a single ip injection at a dose of 20 µg/kg. Brain concentrations are expressed as (mean ± SD, *n *= *5* brain samples from 5 mice)

A pharmacokinetic study was performed for LCA in the mice brain tissues using the validated bioanalytical method after its permeability across the CNS had been affirmed. Brain concentration–time profile for LCA was plotted after injecting the mice ip with LPS (250 µg/kg) for seven consecutive days and a single ip injection of LCA (20 mg/kg) (Fig. 7).

Relevant pharmacokinetic parameters were calculated by non-compartmental analysis, including *T*_max_, *C*_max_, half-life, area under the curve (AUC), apparent volume of distribution (Vz/F), and apparent clearance (Cl/F) (Table 5).

**Table 5:** Pharmacokinetic parameters (mean ± SD, *n* = 5) of LCA in mice brain tissues

Pharmacokinetic parameter	Value (mean ± SD)
*T*_max_ (h)	4.00 ± 0
*C*_max_(µg/mg)	76.91 ± 10.93
*T*_1/2_ (h)	17.13 ± 5.98
AUC_0-t_ (µg/mg).h	571.48 ± 33.52
AUC_0-t/0-∞_	0.51 ± 0.14
MRT_0-t_ (h)	10.50 ± 0.69
Vz/F (mg/kg)	529.74 ± 139.18
Cl/F (mg/kg)/h	17.90 ± 5.33

*T*_*max*_ time to reach maximum brain concentration, *C*_*max*_ maximum brain concentration, *AUC*_*(0–t*)_ area under the concentration–time curve from zero up to a definite time *t*, *AUC *_*(0–∞)*_ area under the concentration–time curve from zero up to infinite time, *T*_*1/2*_ half-life, *Vz/F* volume of distribution, *CL/F* total clearance, *MRT* mean residence time

After a single ip dose, LCA crossed the BBB and reached peak brain levels after 4 h (*T*_max_) with overall high concentrations of 76.91 ± 10.93 µg/mg (*C*_max_). The concentration in the brain tissue remained above 15 µg/mg until 8 h post-injection, demonstrating a good mean residence time (MRT) in the brain tissues of 10.50 ± 0.69 h and a clearance (Cl/F) rate of 17.90 ± 5.33 (mg/kg) h^−1^. Through these results, the reliability and suitability of the established GC–MS/MS method for quantitative analysis were confirmed.

## Discussion

Despite growing evidence of its neuroprotective potential, there is no existing data regarding LCA’s permeability across the BBB, leaving open the possibility that such protective effects might be due to its systemic changes. LCA has demonstrated previously promising effects in both AD and PD in vitro models (Huang et al. 2017a; Chiu et al. 2018; Lin et al. 2020; Guo et al. 2022). However, its efficacy in neurodegenerative disorders and cognitive functions, particularly in vivo, remains scarce. Therefore, this study focused on evaluating the ability of LCA to transverse the CNS and assessing its pharmacokinetics in the brains using the LPS-neuroinflammatory mouse model for the first time in the literature. We first demonstrated LCA’s ability to enhance memory, confirming its potential therapeutic utility. This LPS-neuroinflammatory model, established for its significant memory impairment and AD protein pathology (Lee et al. 2008a; Noh et al. 2014; Zhu et al. 2014; Lykhmus et al. 2015; Zakaria et al. 2016; Abdel-Kader et al. 2021), allowed us to observe the effects of LCA on short-term and recognition memory under neuroinflammatory conditions. Our results signify LCA’s capacity to enhance memory under neuroinflammatory conditions, effectively reversing both the LPS-impaired novel object recognition and the mice’s exploratory activity. Consistent with our findings, cognitive enhancement has been reported previously in vivo after LCA treatment in middle-aged healthy C57BL/6 mice in the absence of a specific disease model (Wu et al. 2021b). Recent studies have further elucidated LCA’s neuroprotective effects, ameliorating ER stress-induced neuronal apoptosis by modulating PERK/eIF2α/ATF4/CHOP signaling in AD models (Fan et al. 2024), enhancing synaptic preservation and reducing NMDA-induced neurotoxicity by inhibiting neuronal necroptosis and glial activation(Kim et al. 2024), and inhibiting the proliferation and invasion of glioma cells by targeting the TLR4/NF-κB signaling pathway (Zhou et al. 2024).

**Fig. 8:**
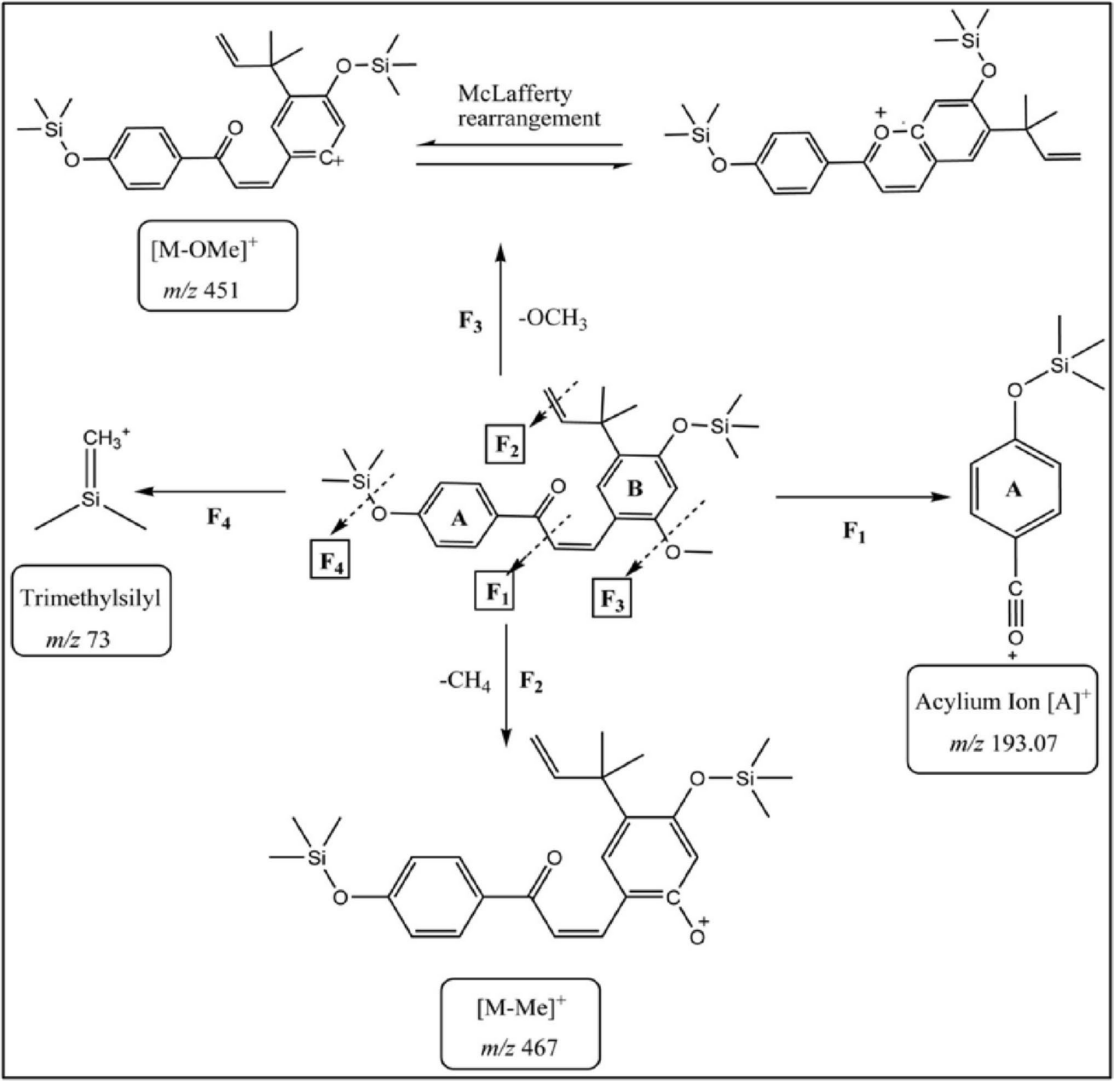
Fragmentation scheme for the 2-TMS LCA derivative based on the abundant *m/z* peaks observed in its mass spectrum

To address the question regarding whether the previously observed memory enhancement effect of LCA is a result of its passage through the BBB, a bioanalytical method was developed to selectively detect LCA in the ip-injected mice’s brain tissue. In this study, we report a newly developed rapid GC–MS/MS analytical method that can detect trace amounts (LOD = 0.14 µg/mL, LOQ = 0.42 µg/mL) of the compound in question, licochalcone A, in the brain tissues of mice. Both IC and LCA have similar chemical structures, making IC a reasonable choice as an internal standard. IC is prenylated chromen-4-one, with 3 hydrogen bond donors (-OH) groups, and LCA is a diphenyl-2-propen-1-one with 2 hydrogen bond donors (-OH) groups. For the separation and detection of LCA, parameters of the developed GC–MS/MS method were optimized. In our study, the oven temperature program achieved heightened separation power and a short analysis timeframe, typically estimated in 15.6 min (Supplementary FigureS1). Hence, our developed method stands within the few rapid GC–MS methods for biological sample analysis reported in the literature (Zhang and Zuo 2004; Shen et al. 2007; Isobe et al. 2011; Xiao et al. 2014). The MS/MS parameters were optimized to achieve high selectivity and sensitivity in detecting the LCA in the brain matrix. Since the GC–MS characterization for LCA has not been reported before in the literature, thus, LCA was preliminarily screened to capture its RT, spectral data, and fragmentation patterns. LCA has a nominal molecular weight of 338.15 Da and an elemental formula corresponding to C_21_H_22_O_4_, possessing two phenolic hydroxyl groups (-OH). During the derivatization reaction, BSTFA was used to introduce two TMS groups (Si(CH_3_)_3_, 73 units) replacing the two hydroxyl hydrogens, thereby converting LCA into its 2-TMS derivative (*D*). The ChemDraw analysis and nominal mass calculation unveiled that the 2-TMS LCA derivative has a molecular weight of 482.23 Da and a molecular formula corresponding to C_27_H_38_O_4_Si_2_. Two distinct eluting peaks denoted as *D*_1_ (at 10.6 min) and *D*_2_ (at 11.2 min), in the generated total ion chromatogram (TIC), were identified as the 2-TMS LCA derivative (Supplementary FigureS2). Since the mass spectra for both D_1_ and D_2_ are identical, they were determined to be *Z*- and *E*-isomers of the 2-TMS LCA, consistent with previous NMR analysis for LCA isomerization (Huang et al. 2017b). The *Z*-isomer peak (*D*_1_) was not included in our analysis because it appeared inconsistently across samples, often falling below the limit of quantification. Although LCA can undergo light-induced *E → Z* isomerization (Huang et al. 2017b), the *Z*-isomer was scarcely detectable under our light-protected protocol. Regarding the isomer-specific pharmacokinetics and activity, to the best of our knowledge, no studies have investigated the pharmacokinetics or biological activity of *E*- versus *Z*-LCA. For chalcones broadly, the *E-*isomer is thermodynamically favored and predominates under physiological conditions, whereas the *Z*-isomer is typically generated ex vivo by light exposure and may show target-specific effects in isolated assays. Given the negligible presence of (*D*_1_) under controlled conditions and the lack of data on *Z-*LCA in vivo, we are confident that quantifying only the *E*-isomer (*D*_2_) accurately reflects LCA exposure in our study. Future investigations using highly sensitive methods could explore any unique properties of the *Z*-isomer. The LCA identification was grounded on the presence of characteristic fragments present in the generated + EI mass spectrum. The fragment at *m/z* 193 is a prominently abundant fragment ion of the acylium ring [A] + corresponding to the p-trimethysilyloxy-benzoyl ion. The acylium ion fragment of the A-ring is characteristic of the chalcone structures and has been reported before for LC–MS with CID analysis of LCA (Huang et al. 2017b) and for GC–MS analysis under EI conditions for substituted chalcone structures (Raza et al. 2016). Moreover, a loss of a methyl group [M–Me] + indicated by *m/z* 467 fragment that has been described before using EI fragmentation at 70 eV for chalcones (Parmar et al. 1993). Finally, the base peak at *m/z* 451, reflecting the removal of the ortho-methoxy group from the parent ion [M-OMe] +, is consistent with previous GC–MS analysis of ortho-methoxy-substituted chalcones yielding a significant or a base peak by McLafferty-type gas phase rearrangement (Raza et al. 2016; Susanti and Setyowati 2019). A proposed fragmentation scheme for the 2-TMS LCA derivative has been established based on the previously discussed abundant fragment peaks observed in its mass spectrum (Fig. 8). The identity of the 3-TMS IC derivative was based on the presence of *m/z* 569 as a base peak in its EI full scan mass spectrum, corresponding to the loss of a –CH3 group (15 Da) from the 3-TMS IC parent molecule of 584 Da. This base peak has been reported before as the chosen monitoring ion for the GC–MS analysis of the 3-TMS IC derivative (Shen et al. 2007).

MRM transitions for both LCA and IC derivatives have been established and optimized for the first time in this study, reporting both the qualifier and quantifier transitions (Table 2). To guarantee high selectivity, particularly in the complex brain matrix intended, the three transitions for the target analyte and IS were monitored using MSD in time-segmented MRM mode.

To verify LCA’s capacity to cross the BBB of the LPS-neuroinflammatory mouse model and to demonstrate the utility of the developed procedure, brain samples from the LCA-injected mice were analyzed. While our results provided the first evidence of LCA’s permeability across the BBB, exhibiting its neuroprotective effects inside the CNS, further studies are needed to conclusively determine whether the detected levels represent true parenchymal penetration or reflect residual vascular content. Although several washing steps were implemented, our current protocol includes brain vasculature, so measured LCA levels may partly reflect residual blood. In vivo perfusion was avoided to prevent anesthesia-related confounding and reduce animal use. Future studies will overcome this limitation by separating vascular and parenchymal fractions using dextran density-gradient centrifugation or immunolabeled FACS sorting, allowing for more accurate assessment of LCA’s CNS distribution. Nonetheless, these findings open new avenues to further investigate its underlying signaling pathways in the CNS. Notably, LCA successfully passed the BBB regardless of whether the mice had undergone LPS pre-treatment or had an unaltered BBB. Quantitative analysis further revealed the greater permeation extent of LCA in mice pre-treated with LPS than those only receiving LCA injections. Although LPS is known to compromise the integrity of the BBB, however, this is recapitulated in neurodegenerative brains where there is an increase in vascular permeability coupled with a reduced expression of BBB tight junction proteins (Wu et al. 2021a; Andjelkovic et al. 2023). After permeability confirmation, pharmacokinetic profiling for LCA in the mice’s brain tissues was performed to have a better understanding of its pharmacological potential. Although LCA has various biological functions, little research has been conducted regarding its pharmacokinetics and analysis in brain tissues. Pharmacokinetic studies revealed LCA’s low oral bioavailability (3.3%) due to the hepatic first-pass effect (Weng et al. 2019), while innovative delivery systems like liposomes and SMEDDS have been shown to enhance its plasma distribution and oral bioavailability in conditions such as chronic renal failure and hyperuricemia (Zhu et al. 2021; Liu et al. 2022). Here, we report for the first time the brain concentration–time profile for LCA to provide insights into how LCA behaves once it enters the brain. The profiling was performed in the context of LPS pre-treatment, which is relevant for understanding how inflammatory conditions might affect the pharmacokinetics of LCA. The PK analysis for LCA in plasma by Weng et al. demonstrated a *C*_max_ of 3173.5 ± 483.0 ng/mL and a *T*_max_ of 0.0833 h after intravenous administration, and a *C*_max_ of 60.7 ± 13.9 ng/mL and a *T*_max_ of 0.5 h after oral administration. This was after a single LCA oral (15 mg/kg) and intravenous (5 mg/kg) dose administration. In our study, LCA reached a peak brain concentration of 76.91 ± 10.93 µg/mg with a *T*_max_ of 4 h after a single intraperitoneal dose of 20 mg/kg. This indicates a longer *T*_max_ and higher brain tissue concentration in our study compared to the blood concentrations by Weng et al. The higher brain concentrations of LCA observed in the LPS model likely reflect cumulative exposure from repeated dosing and possible alterations in LCA distribution and metabolism under inflammatory conditions, which were not captured in the single-dose PK study. While the 20 mg/kg dose was used here to demonstrate CNS delivery and initial behavioral efficacy of LCA, it does not address possible non-linear PK or dose-dependent behavioral effects. Therefore, dose-ranging studies using doses spanning below and above 20 mg/kg will be included in future studies to establish dose–response relationships, PK linearity, and safety margins, thereby strengthening our understanding of LCA’s therapeutic window.

Interpreting the mechanism of transport of the flavonoids into the BBB is often challenging, specifically regarding the metabolic form that imparts the flavonoids such permeability. Using SwissADME and admetSAR 1.0, we evaluated the BBB permeability potential of LCA and its major metabolites in silico. The parent aglycone form of LCA demonstrated high lipophilicity (cLogP ≈ 4.9) and a low topological polar surface area (TPSA = 66.8 Å2), both of which are below established thresholds for passive BBB permeation (Van De Waterbeemd et al. 1998). Consistently, the in silico models predicted a positive BBB permeability for LCA (0.58 probability according to the Boiled Egg model in SwissADME and admetSAR), suggesting that passive diffusion is a likely mechanism for CNS entry of the parent compound. However, the predominant forms of LCA in vivo are the glucuronide metabolic forms as reported in several studies (Nadelmann et al. 1997a, b; Huang et al. 2017b). In other words, LCA metabolites circulating in the bloodstream are more hydrophilic and more susceptible to the efflux transporters, excluding their ability to penetrate the CNS. Nevertheless, the permeability of glucuronide metabolites for flavonoids and polyphenols even with the presence of P-gp efflux transporters has been confirmed before (Youdim et al. 2003; Figueira et al. 2017). However, our in silico analysis of the glucuronide conjugates of LCA exhibited significantly higher polarity, with a TPSA of 125.68 Å2 and a reduced logP of 3.90. These properties, combined with a predicted negative BBB permeability (− 0.8) and probable P-glycoprotein (P-gp) substrate status, suggest that glucuronidation limits CNS penetration. Several studies have concluded that certain metabolic forms of flavonoids, with higher calculated logP values, can penetrate the CNS. These permeable metabolic structures often result from the brain-blood interface metabolizing enzymes, potentially generating novel metabolites. The metabolites with both methylation and sulfation showed higher calculated logP and BBB permeation (Figueira et al. 2017). Additionally, new metabolic derivatives of (+)-catechin and (−)-epicatechin were produced using human brain capillary endothelial cells (Faria et al. 2011). Another study identified two unique metabolites, comprising both methyl and sulfate groups, in the brain tissue out of 51 upon LC–MS profiling (+)-catechin metabolites in rats (Liang et al. 2014). LCA has been reported to undergo both sulfation and methylation metabolic pathways upon analysis of human hepatocyte metabolites (Huang et al. 2017b). Interestingly, the in silico evaluation of the sulfated LCA metabolite displayed a moderate logP of 4.30, a TPSA of 118.51 Å2, and a surprisingly positive predicted BBB permeability (0.83), indicating that despite its polarity, sulfation may not entirely prevent brain access. The methylated catechol metabolite also showed enhanced BBB permeability potential, with a high logP of 4.87, a moderate TPSA of 75.99 Å2, and a predicted permeability probability of 0.525, further supporting the role of methylation in improving CNS availability. Moreover, several studies suggest the permeability of N-acetylcysteine across the BBB in both humans and rodents with no need for active transport (Farr et al. 2003; Bavarsad Shahripour et al. 2014; Katz et al. 2015). Interestingly, LCA N-acetyl-l-cysteine conjugates were previously formed in vitro by Nadelmann et al., indicating these conjugates can result from a potential metabolic pathway for the compound (Nadelmann et al. 1997b), but the in silico evaluation showed negative predicted BBB permeability. Hence, the question remains about the specific metabolic form of LCA that can cross the blood–brain barrier, which is crucial for better understanding the transporters involved in this process and for designing potential drugs for neurodegenerative diseases. Future research will focus on developing an extraction protocol that preserves the structure of LCA metabolites in biological samples to identify and quantify specific LCA metabolites within the CNS. Additionally, in vivo studies using capillary-depleted brain fractions will help distinguish between vascular retention and true parenchymal accumulation.

## Conclusion

In conclusion, our study sheds light on the neuroprotective potential of licochalcone A in enhancing memory. Using an LPS-neuroinflammatory mouse model, we demonstrated LCA’s ability to enhance short-term and recognition memory through behavioral tests. Our study also introduced a novel GC–MS/MS analytical method, optimized, and validated for detecting low levels of LCA in complex brain tissues. The reliability of the developed analytical method was demonstrated in qualitative and quantitative analysis of LCA in mice brain tissues along with pharmacokinetics profiling. Our findings validate LCA permeability across the BBB, paving the way for future studies to unravel its underlying mechanisms in the CNS.

## Supplementary Information

Below is the link to the electronic supplementary material.

ESM 1 (DOCX 1.11 MB)

## Data Availability

All source data for this work (or generated in this study) are available upon reasonable request.
