# Supplementary material for: Detection and pharmacokinetics of licochalcone A in brains of neuroinflammatory mouse model
Source: Naunyn Schmiedebergs Arch Pharmacol. 2025 Sep 8;399(2):2663–82. doi: 10.1007/s00210-025-04579-w (PMC12901191; doi:10.1007/s00210-025-04579-w)
Supplement: Supplementary file 1 — (DOCX 1.11 MB) [file 210_2025_4579_MOESM1_ESM.docx]

**Supporting Information**


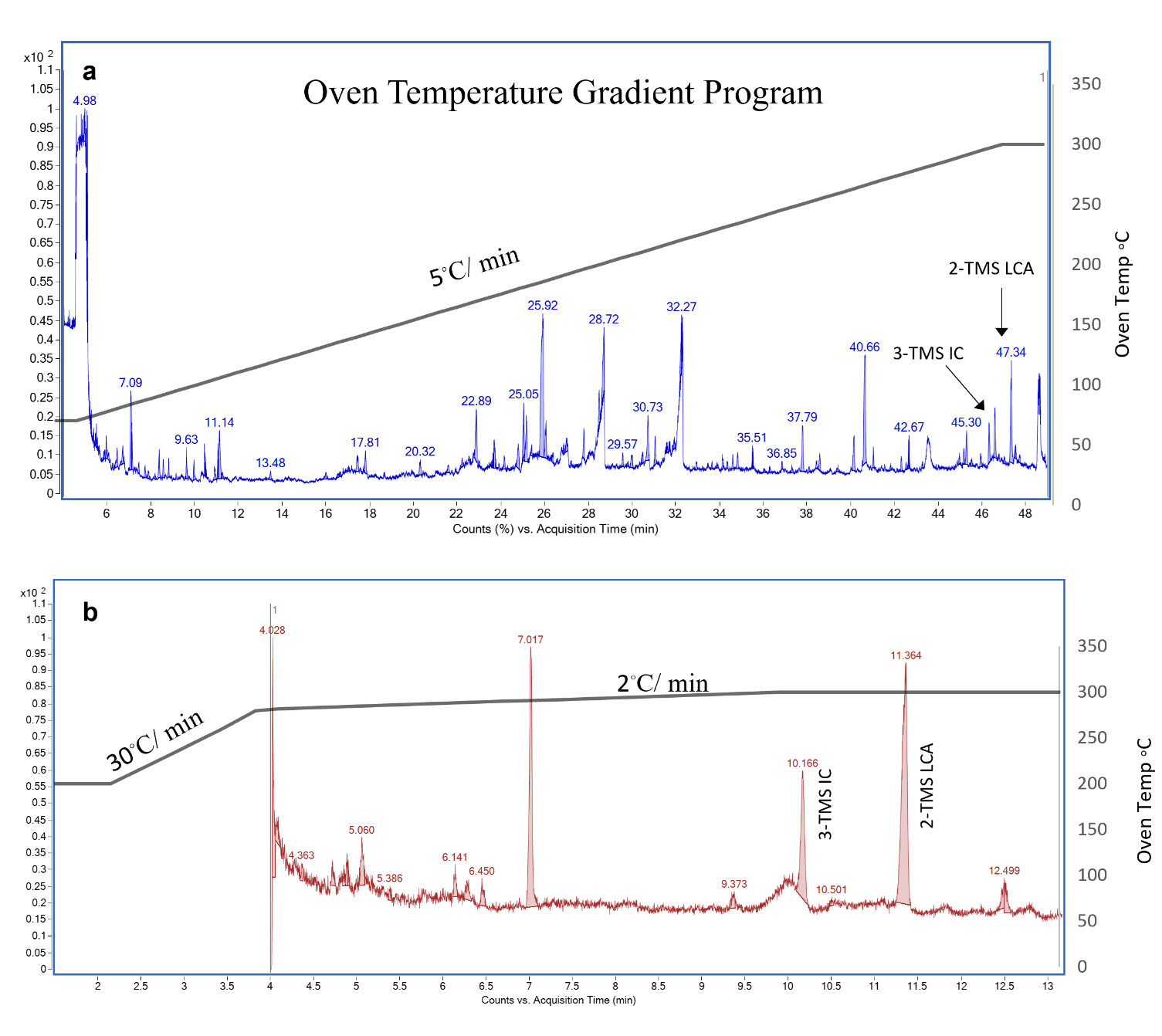


**Figure S1** The oven temperature gradient was optimized for good separation and short analysis time. (a) total run time of 49 min with LCA and IC appearing at 47.3 and 46.3 min, respectively. (b) total runtime of 15 min after temperature program optimization and fast separation of LCA and IC at 11.3 and 10.1 min, respectively


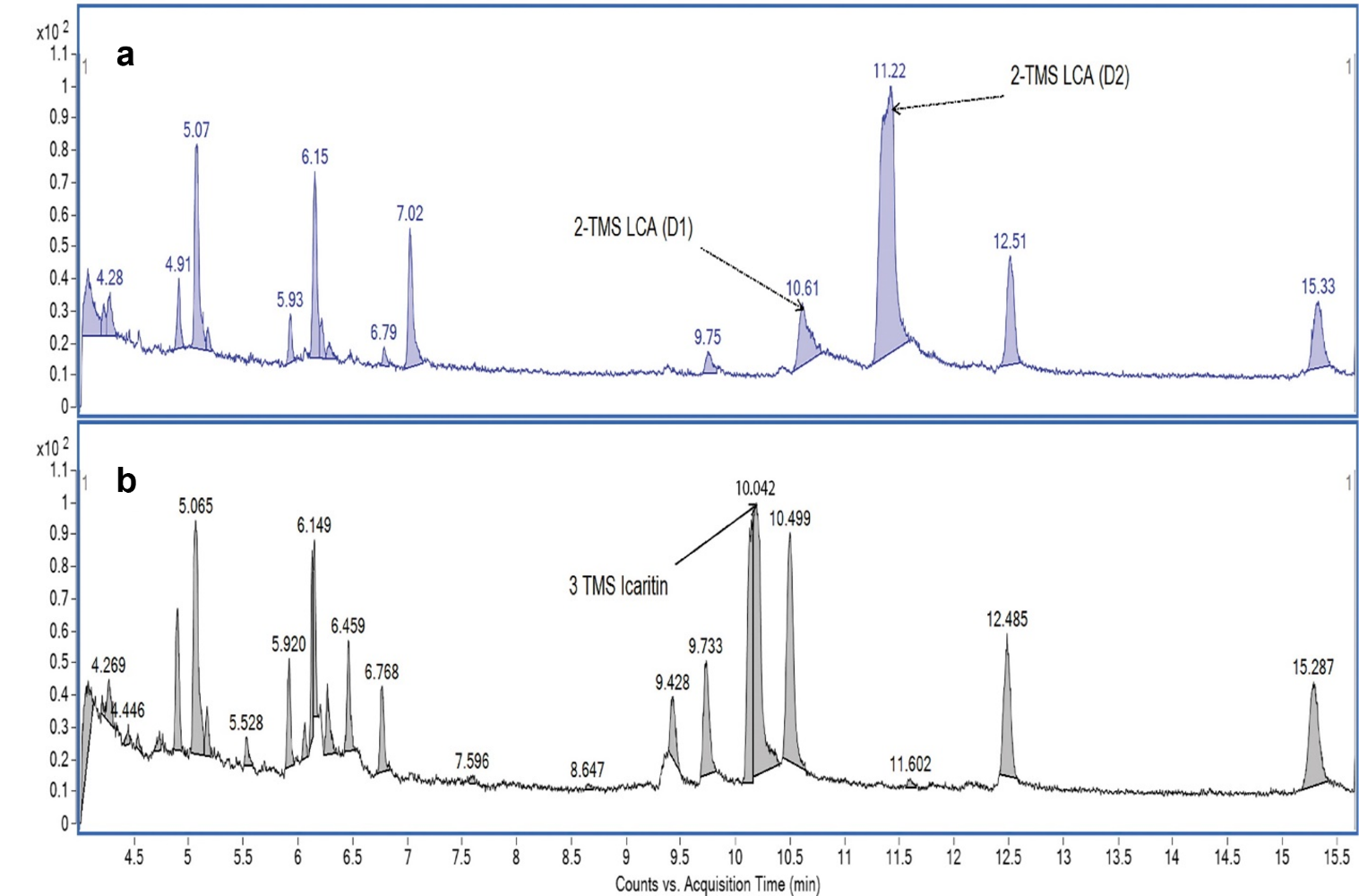


**Figure S2** +EI full scan chromatograms for derivatized reference standards at 0.5 µg/ml and 0.05 µg/ml. (a) Full scan chromatogram for 2-TMS LCA derivative appeared at two adjacent peaks at 10.61 min and 11.22 min denoted as D_1_ and D_2_ (b) Full scan chromatogram for 3-TMS IC derivative appeared at 10.04 min


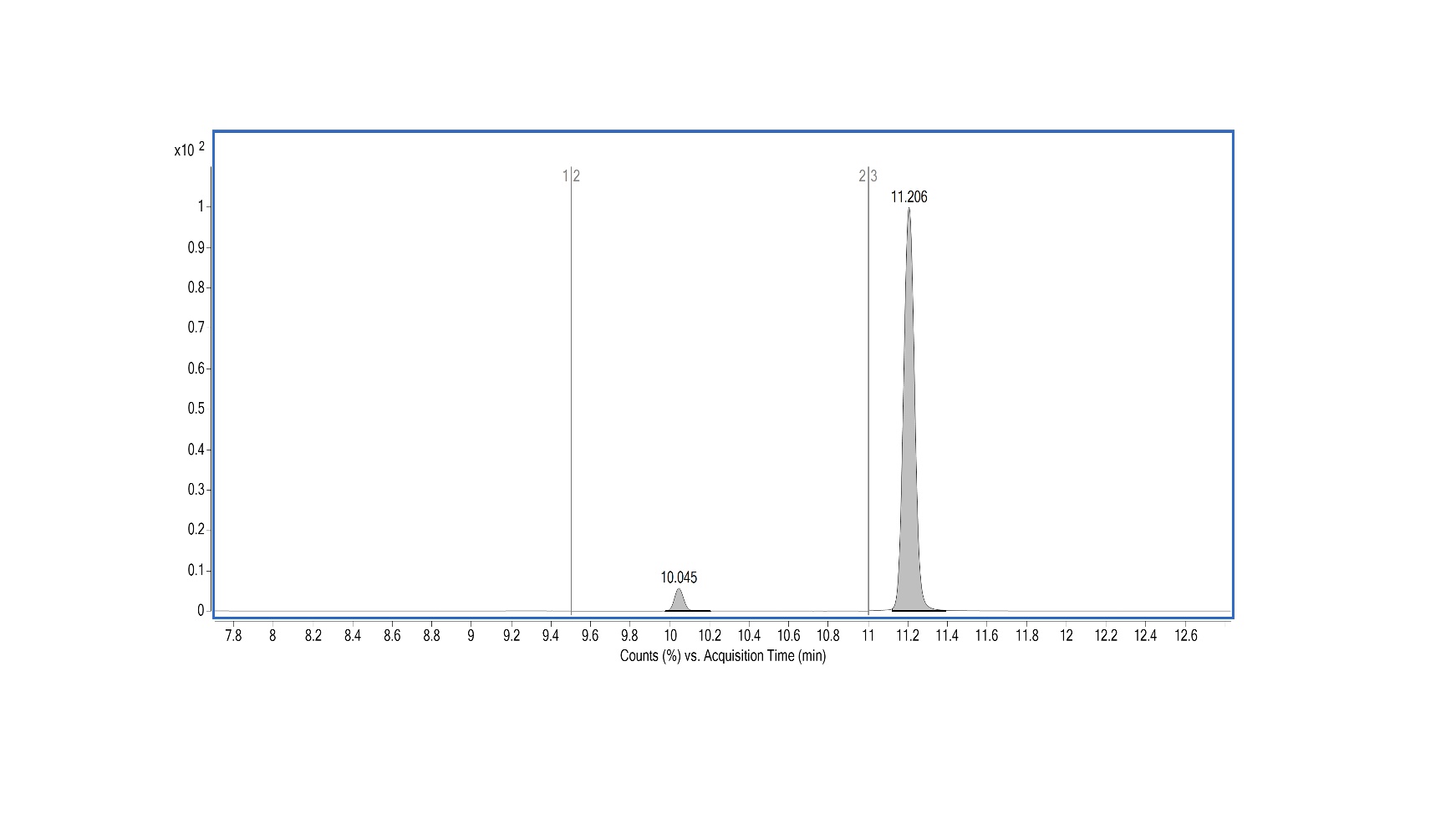


**Figure S3** +EI-TIC chromatogram in time-segmented MRM mode with 2-TMS LCA specific transitions from 4-9.5 min, 11-15.6 min, and 3-TMS IC derivative specific transitions from 9.5- 11 min


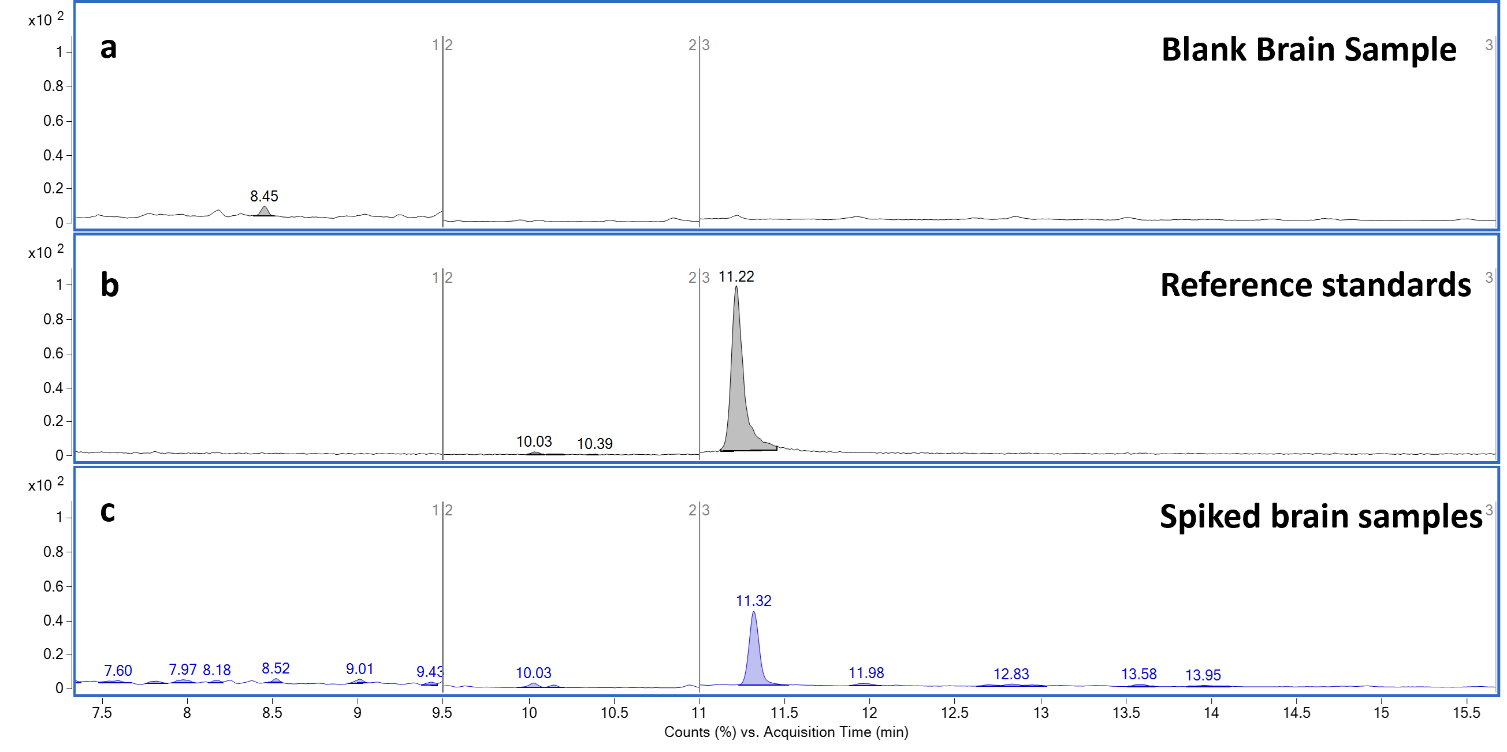
 **Figure S4** Identity confirmation of LCA (analyte) and IC (IS) using reference standards at concentrations of 0.5 µg/ml and 0.05 µg/ml, respectively (a) Blank Brain samples confirmed to be true negative of LCA and IC (b) Spiked standards on pyridine solvent (c) Spiked standards on brain samples


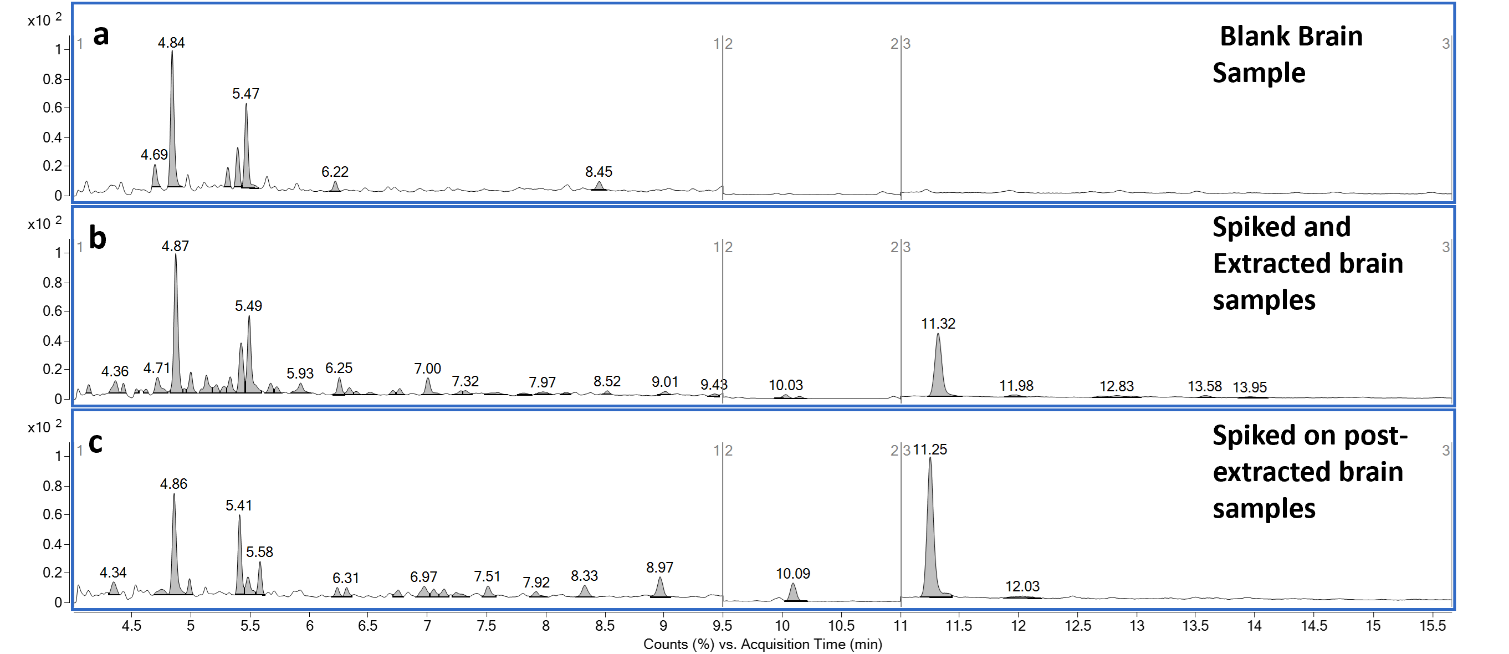


**Figure S5** Selectivity of the bioanalytical method represented by +EI-MRM Chromatograms of (a) representative blank brain sample (b) spiked and extracted standards on blank brain sample at LOQ (c) spiked on post-extracted blank brain sample at LOQ


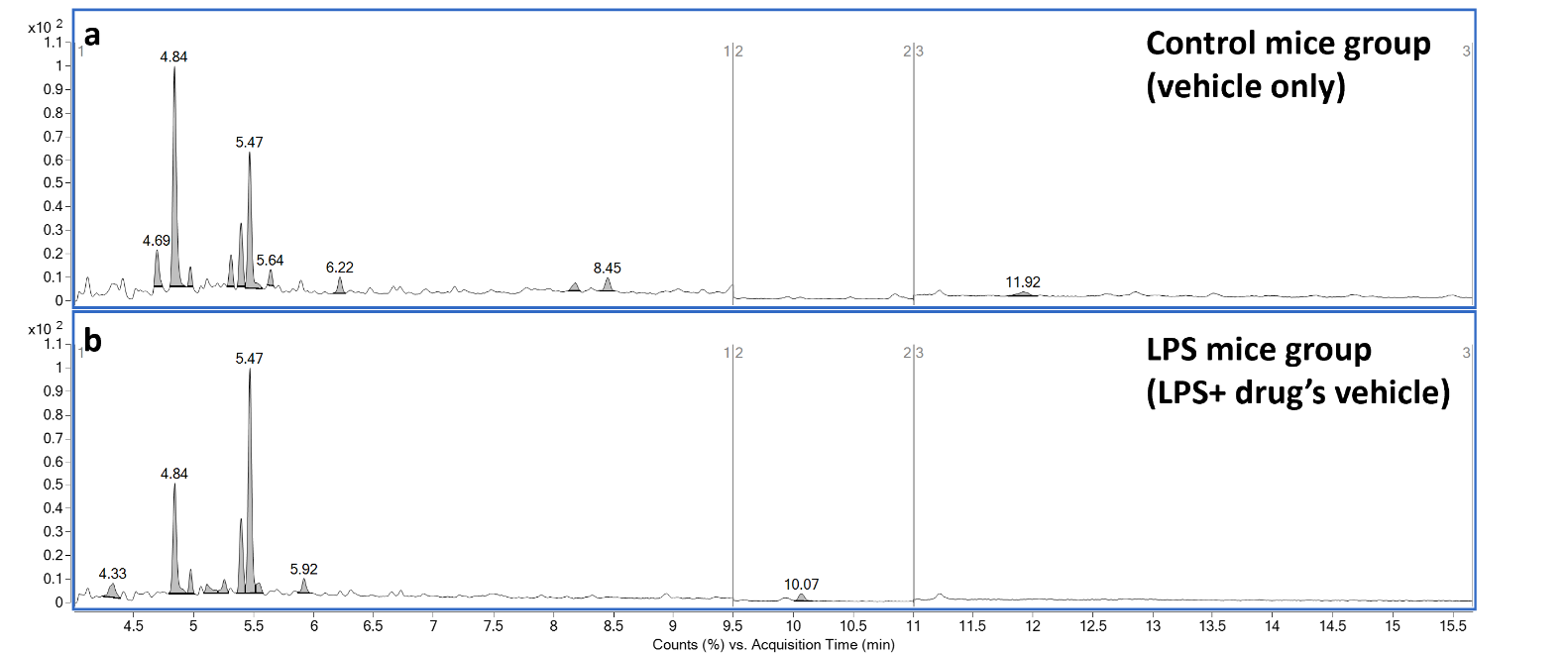


**Figure S6** +EI-MRM chromatogram for the LPS neuro-inflammatory mice groups (a) Control mice group (b) LPS mice group, operating MSD in time-segmented MRM mode. Both were confirmed to be true negatives of the analyte

**Table S1** Calibration runs of LCA in brain matrix for linearity test. Data expressed as (mean ±SD, n=9, 3 replicates per day)

| Calibrator Spiked concentration (µg/ml) | Measured concentration (µg/ml) | Precision (CV%) | Mean Accuracy Day 1 | Mean Accuracy Day 2 | Mean Accuracy Day 3 |
| --- | --- | --- | --- | --- | --- |
| 0.5 | 0.48 ±0.03 | 5.39 | 94.59 ±2.91 | 96.35 ±7.27 | 96.84 ±6.44 |
| 0.75 | 0.68 ±0.03 | 3.85 | 89.54 ±2.70 | 90.04 ±4.43 | 93.84 ±2.39 |
| 1.5 | 1.47 ±0.09 | 6.39 | 102.28 ±0.62 | 92.90 ±6.66 | 97.98 ±6.72 |
| 5 | 4.48 ±0.15 | 3.27 | 87.59 ±2.94 | 91.41 ±3.11 | 89.84 ±2.21 |
| 10 | 11.04 ±0.18 | 1.61 | 111.83 ±1.38 | 109.40 ±2.41 | 110.00 ±0.32 |
| 20 | 19.67 ±0.10 | 0.51 | 98.29 ±0.66 | 98.40 ±0.75 | 98.40 ±0.06 |
| Slope (mean ±SD) | 8.51 ±0.12 | | | | |
| y-intercept (mean ±SD) | 1.89 ±0.36 | | | | |
| R^2^ (minimum-maximum) | 0.9912 -0.9976 | | | | |
